# An ensemble approach using multidimensional convolutional neural networks in wavelet domain for schizophrenia classification from sMRI data

**DOI:** 10.1038/s41598-025-93912-7

**Published:** 2025-03-25

**Authors:** Tamilarasi Sarveswaran, Vijayarajan Rajangam

**Affiliations:** 1 School of Electronics Engineering, Vellore Institute of Technology, Chennai, India; 2 Centre for Healthcare Advancement, Innovation and Research, School of Electronics Engineering, Vellore Institute of Technology, Chennai, India

**Keywords:** sMRI, DWT, Schizophrenia, 1D-CNN, 2D-CNN, 3D-CNN, Network models, Magnetic resonance imaging, Engineering, Mathematics and computing

## Abstract

Schizophrenia is a complicated mental condition marked by disruptions in thought processes, perceptions, and emotional responses, which can cause severe impairment in everyday functioning. sMRI is a non-invasive neuroimaging technology that visualizes the brain’s structure while providing precise information on its anatomy and potential problems. This paper investigates the role of multidimensional Convolutional Neural Network (CNN) architectures: 1D-CNN, 2D-CNN and 3D-CNN, using the DWT subbands of sMRI data. 1D-CNN involves energy features extracted from the CD subband of sMRI data. The sum of gradient magnitudes of CD subband, known as energy feature, highlights diagonal high frequency elements associated with schizophrenia. 2D-CNN uses the CH subband decomposed by DWT that enables feature extraction from horizontal high frequency coefficients of sMRI data. In the case of 3D-CNNs, the CV subband is used which leads to volumetric feature extraction from vertical high frequency coefficients. Feature extraction in DWT domain explores textural changes, edges, coarse and fine details present in sMRI data from which the multidimensional feature extraction is carried out for classification.Through maximum voting technique, the proposed model optimizes schizophrenia classification from the multidimensional CNN models. The generalization of the proposed model for the two datasets proves convincing in improving the classification accuracy. The multidimensional CNN architectures achieve an average accuracy of 93.2%, 95.8%, and 98.0%, respectively, while the proposed model achieves an average accuracy of 98.9%.

## Introduction

Schizophrenia (SZ) is a complicated brain disease with symptoms that include hallucinations, delusions, disorganized thinking, and a flat mood^1^. Positive symptoms such as delusions and hallucinations coexist with negative symptoms such as decreased emotional expression and motivation^2^. Many schizophrenics have enlarged ventricles in their brains, indicating structural problems. There is evidence of decreased brain volume in the areas responsible for cognition and emotion^2^. People suffering from schizophrenia have decreased communication between their brain hemispheres^3^. Previous studies have shown that schizophrenia affects the temporal and anterior lobes of the hippocampus in the brain. This condition is related to an increase in cerebrospinal fluid volume (CSF) and a reduction in the volumes of white matter volume (WMV) and gray matter volume (GMV)^3^. Neuroimaging methods, functional and structural modalities^3,4^, play a crucial role in schizophrenia diagnosis since they avail the clinicians with vital information about brain activity and anatomy. The primary purpose of structural neuroimaging techniques is to expose the anatomy and connections of the brain. Structural Magnetic Resonance Imaging (sMRI) is a technique that provides detailed images of brain structures using strong magnetic fields in addition to radio waves^5^. It offers high resolution, thus clinicians can compare numerous brain regions and be able to identify any alterations in size that may be pathological symptoms of SZ^6^.

In the schizophrenia classification using sMRI, several deep learning (DL) models and machine learning (ML) methods have been explored. These techniques use datasets consisting of neuroimaging and clinical records to identify patterns associated with schizophrenia. The multilayered neural networks inherent in DL approaches examine features across numerous layers, boosting their ability to detect subtle and complicated signs of SZ^7^. Significant biomarkers in sMRI include GMV loss and changes in cortical thickness^8^. Additionally, sMRI detects differences in cortical surface area and gyrification patterns^9^. These indicators indicate structural brain abnormalities, which are critical in diagnosing schizophrenia. One-dimensional convolutional neural network (1D-CNN) is highly regarded for their simplicity, computational efficiency, and flexibility to complex one-dimensional input sets. Their shallow architecture makes them easy to train, and deploy. It also provides techniques for detecting temporal patterns and anomalies in brain activity^10^. A two-dimensional convolutional neural network (2D-CNN) is used to detect spatial anomalies in brain imaging data. This is critical for identifying structural brain modifications linked with schizophrenia^11^. Three-dimensional convolutional neural network (3D-CNN) uses kernels with three dimensions: two spatial and one temporal depth^12^. These are well-suited for volumetric data such as functional MRI (fMRI) and MRI, enabling thorough investigation of brain volumes to discover schizophrenia-related aberrations in both structure and function^11,12^. The activation functions ReLU, Sigmoid, and Tanh are important in identifying complex patterns in the brain’s input. The non-linearity of the activation function facilitates SZ categorization at a detailed level by simulating complex relationships within the data^13^. Some solutions to these problems would include centralized learning and data anonymization that could be explored to conduct bigger research efforts and even more complete studies on schizophrenia^14^.

Recent advances in the analysis of sMRI brain volumes have used a variety of machine learning and deep learning approaches to reveal precise data about brain anatomy. Diverse approaches were used to improve the accuracy and specificity of the results. For instance, dVoxResNet and Machine Learning Discriminant Analysis (MLDA)^15^ have already been used to accurately measure the sMRI brain volumes^16^ by segmenting, classifying, and detecting brain structures or abnormalities with high accuracy. Enet-TV classifier has been applied in estimating GMV^17^. The combination of the Inception ResNet model and Support Vector Machine (SVM) is a sophisticated strategy that combines deep learning for feature extraction and machine learning for classification^13^. The use of 3D-CNN for both feature extraction and classification emphasizes the trend of using complicated neural network architectures in brain imaging research^18^. SVM-based explorations of various brain regions have yielded useful discoveries, particularly when assessing cortical thickness and surface area^19,20^. Studies have used SVM to investigate both WMV and GMV and the use of Random Forest (RF) classifier to detect abnormalities based on cortical and geometric properties demonstrates the adaptability of these machine learning technologies^21^. SVM has been used extensively to investigate specific brain subregions such as the amygdaloid and hippocampal areas, as well as GMV^22,23^. Furthermore, use of 3D Convolutional Autoencoder (3D-CAE) model with logistic regression has proven to be useful in diagnostics^24^. SVM-based analysis for GMV and WMV evaluation and voxel-based morphometry integrated techniques has improved our understanding of the anatomy of the brain using advanced computational techniques^25^. These different approaches illustrate the dynamic and fast growing nature of brain imaging research, as each study contributes to a more complex understanding of brain anatomy and its complexities. The enormous size of the schizophrenia dataset requires a more powerful graphics processing unit (GPU) for computations. The computational demands are substantial, resulting in prolonged execution times for dataset-related analysis and operations. This issue is dealt with using downsampled DWT subbands. First-level discrete wavelet transform (DWT) decomposition reduces the size of an image in half for each frequency subband^26^.

The proposed method classifies sMRI images through a multi-stage process using discrete wavelet transform and multidimensional convolutional neural networks. The slices in sMRI are decomposed into four subbands using DWT^26,27^ known as approximate coefficients (CA), vertical coefficients (CV), horizontal coefficients (CH), and diagonal coefficients (CD). The feature extraction from the frequency subbands in wavelet domain tends to provide better classification results by identifying abnormalities in the brain structures related to schizophrenia, such as cortical folding patterns, changes in surface areas, and abnormal curvature of the cortical surface. The DWT subbands are processed using three separate CNNs: 1D-CNN, 2D-CNN, and 3D-CNN, each with a different wavelet coefficient for feature extraction. Collectively, this approach is termed as multidimensional CNNs (MDCNNs). Specifically, energy characteristics are derived from CD subband and 1D-CNN is used for classification. The CH subband is fed into a proposed 2D-CNN to extract useful features for schizophrenia categorization. Similarly, the CV subband is applied to a 3D-CNN model to detect volumetric features associated with schizophrenia. Energy values are observed from the CD coefficients across 88 images out of 192 images in the COBRE dataset, and 176 images out of 192 images in the UCLA & OpenfMRI datasets, which contain important brain regions affected by schizophrenia. Each image of sMRI of a subject has different features from the left and right hemispheres of the brain, such as the temporal cortex, insula, hippocampus, and so on. The features pertaining to SZ are spread across 21 images, where each image is a specific region or a mix of regions. For example, image 11 is correlated with the left temporal cortex, and image 86 is correlated with the right temporal cortex. The energy estimation here is based on the gradient magnitude of the image in each subject. The energy calculation is based on not just the intensity values, but also the neighborhood pixels. As the gradient magnitude increases, the energy value also increases, indicating the abnormalities in sMRI scans. The energy levels are then fed into 1D-CNN, which performs further classification. 2D-CNN is used to examine the spatial properties of all 88 images in a single subject based on the CH subband. 3D-CNN extracts feature from the volume of pixels using neighborhood processing and the CV subband. The results of the MDCNNs are then integrated using a max-voting ensemble technique, which selects the most often predicted category as the final output, increasing classification accuracy and reliability. The performance of the proposed method is measured using accuracy, precision, recall, and F1 score. The effectiveness of the proposed method is analyzed by various metrics and two different datasets. The max-voting ensemble technique is to determine the multidimensional feature extraction and classification output for schizophrenia prediction. In addition, the max-voting method minimizes false positives (FP) in the COBRE dataset and true negatives (TN) in the UCLA & OpenfMRI dataset. The max-voting technique is not just the ensemble method, it integrates the classification outputs obtained from the multidimensional environment using features extracted in DWT domain. The features extracted in multiscale/multiresolution domain are better in revealing local patterns, edges and textures etc. The energy values evaluated from multiscale frequency subbands represent coarse and fine details of the brain regions contributing to SZ classification. This study involves a novel approach that uses multi-resolution analysis and MDCNNs on structural MRI data, demonstrating CNNs’ ability to detect complex patterns associated with schizophrenia. The main contributions are

- DWT subbands for the classification task using MDCNNs architectures. The downsampled DWT subbands reduce the amount of data processed by the networks.
- Investigation of MDCNNs architectures for SZ classification. Preparing sMRI dataset for multidimensional CNN models: Energy feature extraction from the CD subband of sMRI dataset comprising 234 healthy and 177 schizophrenia subjects for training and testing of 1D-CNN, CH subband for spatial fusion-based 2D-CNN and CV subband for volumetric -based 3D-CNN.
- Max-voting to the multiresolution feature extracted classification outputs of MDCNNs for improved accuracy.
- Generalization of the proposed method for two datasets for performance analysis.

This paper is organized as follows: section “Literature review” examines the existing schizophrenia classification studies, focusing on deep learning and standard machine learning methodologies. Section “Dataset description and wavelet decomposition” entails the description of the dataset, preprocessing of sMRI, and the application of DWT for sMRI decomposition. Section “Proposed method and performance analysis” gives the proposed approach, including the selection of subbands for CNN architectures, evaluating the performance of MDCNNs models, analysis of the confusion matrix, and applying max-voting analysis for our proposed Max-Voting based Multidimensional Deep Learning Model (MVMDM). Section “Comparative analysis” analyzes the performance of the proposed model with existing models, focusing on accuracy. Section “Generalization of the MVMDM” investigates the generalization of the proposed method using two datasets by comparing models trained on the Center for Biomedical Research Excellence (COBRE) - University of California, Los Angeles (UCLA) and Open Functional Magnetic Resonance Imaging (OpenfMRI) datasets and vice versa. Section “Limitations and future work” analyzes model limitations and discussed about future work. Section “Conclusion” concludes that customized CNN models perform better in sMRI classification.

**Fig. 1:**
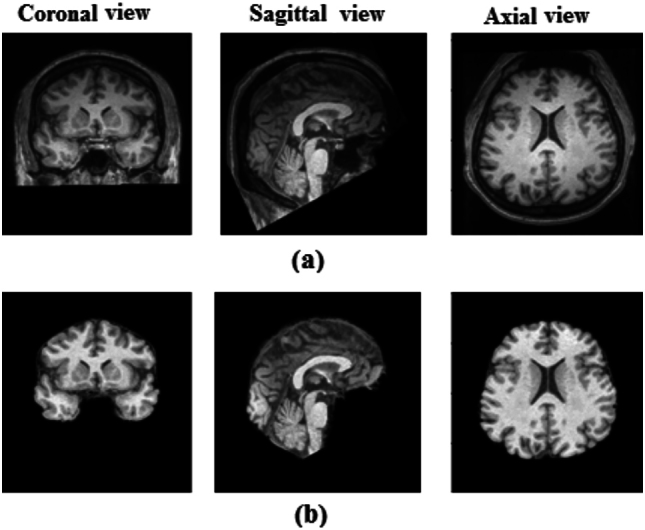
Schizophrenia sMRI image. (**a**) Original image (**b**) preprocessed image.

## Literature review

Table 1 provides a comprehensive literature review on SZ research using sMRI, the dataset, extracted features, and classifier. Chen et al.^1^ have worked on white matter and gray matter features based on the COBRE dataset. SVM classifiers were used to do classification tasks, indicating that machine learning techniques are utilized in order to distinguish between the types of brain tissues. De Moura et al.^15^ concentrated on sMRI brain volume analysis using clinical data. The authors used a method called MLDA for classification. The statistical method, MLDA, is employed to identify structural variations in the brains of clinical groups. Pominova et al.^16^ examined the UCLA dataset, emphasizing sMRI brain volume analysis. The authors used dVoxResNet, a deep-learning network for classification tasks. Robust analysis of structural brain changes is likely made possible by the dVoxResNet architecture to handle volumetric data effectively. De Pierrefeu et al.^17^ focused on examining GMV using. Enet-TV classifier, with two datasets; NMorphCH and COBRE. The combination of total variation regularization and elastic net regularization, Enet-TV is actually well compatible with brain imaging highly dimensional data.

**Table 1:** Literature review on schizophrenia using sMRI data.

References	Dataset	Feature	Classifier	Accuracy (%)
Chen et al.^1^	COBRE	GMV and WMV	SVM	85.9
De Moura et al.^15^	Clinical data	sMRI brain volume	MLDA	73.0
Pominova et al.^16^	UCLA	sMRI brain volume	dVoxResNet	73.9
De Pierrefeu et al.^17^	NMorphCH, COBRE	GMV	Enet-TV	91.7
Yamamoto et al.^19^	Clinical data	Bilateral medial frontal cortex, superior temporal cortex, insula, occipital cortex, cerebellum, thalamus	SVM	70.0
Xiao et al.^20^	Clinical data	Cortical thickness and surface area	SVM	85.0
Lu et al.^21^	Clinical data	GMV and WMV	SVM	88.4
Guo et al.^22^	COBRE	Amygdaloid and hippocampal subregions	SVM	81.2
Dwyer et al.^23^	MRN, COBRE	GMV	SVM	81.2
Yamaguchi et al.^24^	COBRE, Kyoto University	Voxel intensity values	3D-CAE and LR	NA [diagnosis]
Tas et al.^25^	Clinical data	Voxel-based morphometry	SVM	78.2
Hu et al.^28^	IMH, NUSDAST	Various features	Inception ResNet and SVM	70.9
Liu et al.^29^	Clinical data	Cortical and geometric features	RF	NA [detects abnormalities]
Liu et al.^30^	Clinical data	Cortical thickness	SVM	88.7
Latha et al.^31^	NA-MIC	Texture features	Fuzzy SVM	90.0
Pinaya et al.^32^	Clinical data	Cortical thickness and anatomical volumes	DBN-DNN	73.6

Yamamoto et al.^19^ used an SVM classifier to analyze data from different parts of the brain: thalamus, bilateral medial frontal cortex, superior temporal cortex, insula, occipital cortex, and cerebellum. Xiao et al.^20^ examined cortical thickness and surface area using clinical data. The authors used an SVM classifier, demonstrating the application of machine learning techniques for the classification of anatomical brain traits linked to clinical disorders. Lu et al.^21^ employed clinical data to analyze the volumes of the white and gray matter. SVM was utilized for classification tasks, which probably made it easier to identify structural differences between clinical populations and healthy controls. Guo et al.^22^ looked at the hippocampus and amygdaloid sub-regions using the COBRE dataset. SVM was used to identify structural abnormalities in particular brain regions linked to psychiatric diseases. Dwyer et al.^23^ used an SVM classifier to examine GMV using the Mind Research Network (MRN) and COBRE datasets. SVM was used for classification and achieved an accuracy of 81.2%. Yamaguchi et al.^24^ used a 3D-CAE and logistic regression (LR) for classification analysis on the COBRE and KYOTO university datasets. Designed to learn hierarchical representations of 3D data such as sMRI images, the 3D-CAEs facilitate efficient feature extraction for LR-based classification tasks. Research done with the DWT in structural MRI for schizophrenia categorization demonstrates the effectiveness of biomarkers linked with the disease.

Tas et al.^25^ analyzed data based on clinical information using voxel-based morphometry (VBM). By conducting voxel-wise analysis of the images of the brain, SVM was used in classification tasks to determine structural differences between clinical populations and healthy controls. Hu et al.^28^ utilized the Inception ResNet architecture in analyzing the Institute of Mental Health (IMH) and Northwestern University Schizophrenia Data and Software Tool (NUSDAST) datasets. Inception ResNet is well-known for processing high-resolution images with good efficiency. Liu et al.^29^ examined cortical and geometric aspects in clinical data. The authors used an ensemble learning approach called RF classifier, which is a flexible algorithm that can handle complicated feature interactions and high-dimensional data.

Liu et al.^30^ used clinical data for cortical thickness analysis. The authors used an SVM classifier to identify cortical abnormalities linked to medical disorders. Latha et al.^31^ concentrated on textural features collected from brain images through analysis of the National Alliance for Medical Image Computing (NA-MIC) dataset. For classification tasks, the authors used a Fuzzy SVM classifier, a kind of SVM appropriate for managing ambiguity in feature representations. Pinaya et al.^32^ used a deep neural network (DNN) and deep belief network (DBN) for cortical thickness and anatomical volumes to classify schizophrenia, achieving notable results in distinguishing between schizophrenia patients and healthy controls. Each of the research uses different number of attributes, datasets, and classifiers to assess changes that occur in brain structures along with abnormalities, therefore complementing each other in developing neuroimaging analysis.

## Dataset description and wavelet decomposition

MRI datasets, presented in Table 2 were gathered from publicly available sources, notably the UCLA Consortium for Neuropsychiatric Phenomics LA5c Study database, which contains data from 50 schizophrenia-affected people and 130 healthy controls^16,33,34^. OpenfMRI has data from 41 healthy volunteers and 58 people with schizophrenia available in sMRI and fMRI formats^35^. Furthermore, the COBRE dataset includes data from 74 healthy people and 72 people diagnosed with schizophrenia^36,37^.

### sMRI preprocessing

**Fig. 2:**
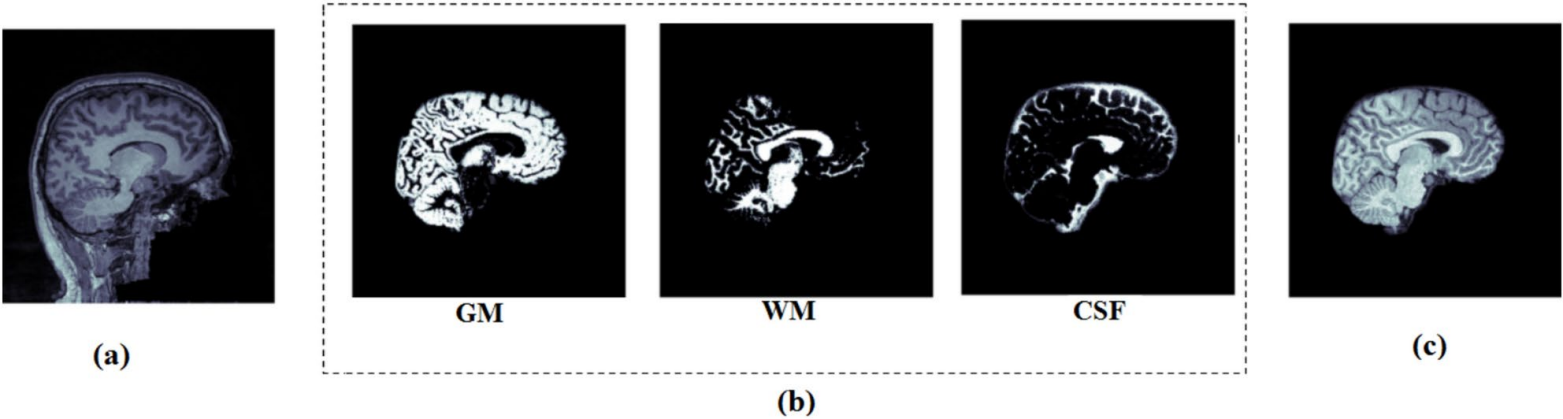
Preprocessing of sMRI (**a**) original sMRI (**b**) segmentation of sMRI into gray matter, white matter, and CSF (**c**) Bias corrected and skull stripped image.

Structural MRI scans shown in Fig. 1 capture the structural properties of the brain and are used for various analyses, such as brain anatomy research and anomaly diagnosis^38^. Several factors contribute to the degradation in sMRI, including motion artifacts, stabilization period, and eventual signal saturation or decay. Bias correction (BC) and bias regularization (BR) address the presence of a low-frequency but smooth bias field signal in MRI, which can distort images obtained by older MRI machines^39^. Skull stripping, also known as brain extraction (BE), is one of the most important preprocessing steps for removal of non-brain material from MR brain images. As shown in Fig. 2, this step is highly essential in the analysis of brain images. It facilitates automated skull stripping and reduces processing time while increasing data accuracy. In this study, the sagittal section is used.

**Table 2:** Description of MRI Datasets.

Datasets description	UCLA LA5c study	OpenfMRI	COBRE
Scanner field strength	3T	3T	3T
Modality	sMRI/fMRI	sMRI/fMRI	sMRI/fMRI
Acquisition year	2016	2017	2017
Age mean ± SD (years)	31 ± 10	29 ± 8	32 ± 12
Shape of sMRI	(176,256,256)	(176,256,256)	(192,256,256)
No. of normal scans	130	41	74
No. of schizophrenia scans	50	58	72
No. of images taken for classification	167	99	145
Total no.of subjects with shape	411(88,256,256)		
No. of images taken for training/testing	(329/82)		

**Algorithm 1:**
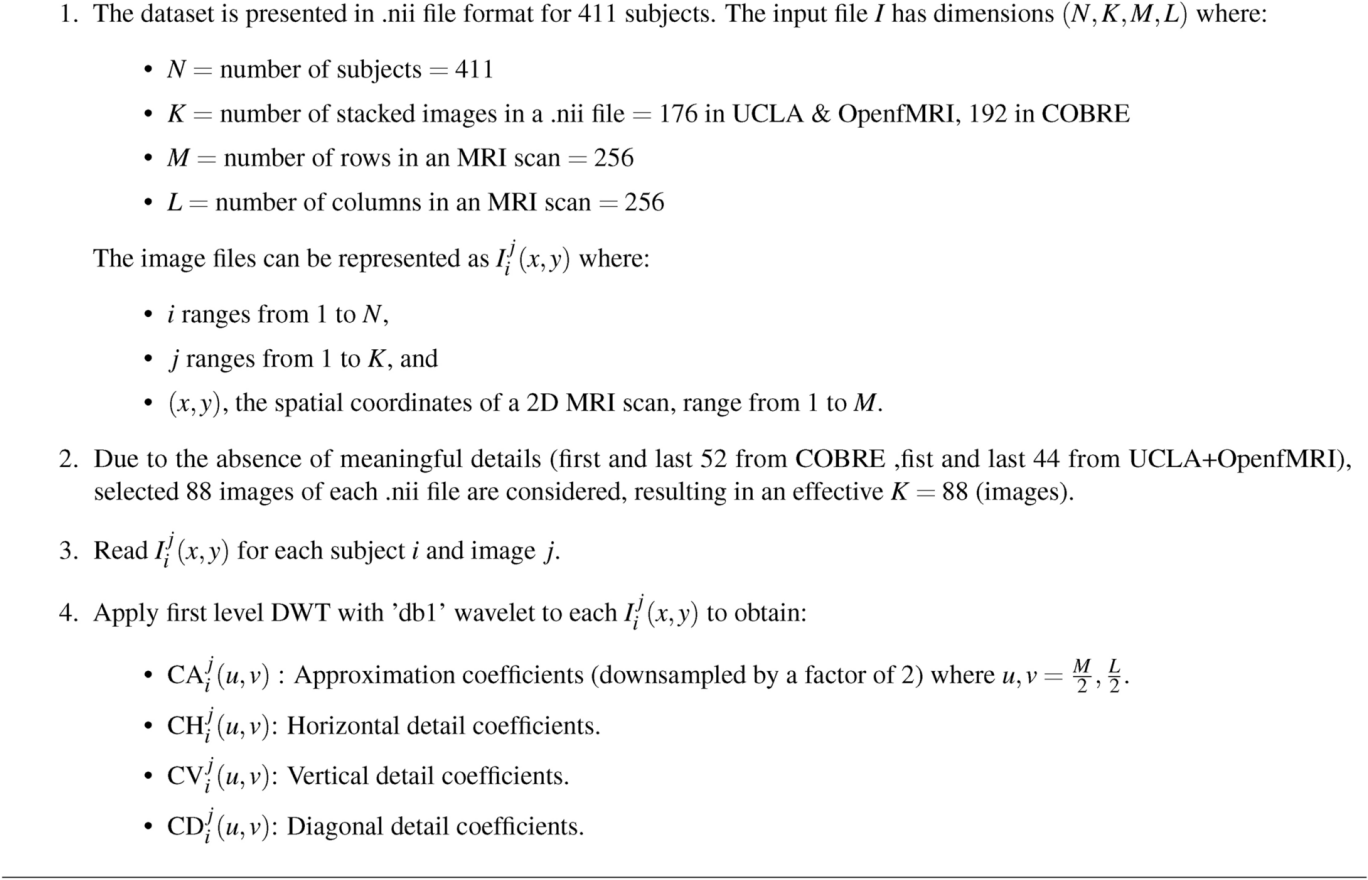
DWT decomposition of sMRI

### sMRI wavelet decomposition

DWT decomposes an image into four subbands: CA, CH, CD, and CV^26,27^. This decomposition is accomplished using two sets of functions: wavelet functions $\psi (t)$ and scaling functions $\Phi (t)$. The DWT coefficients are derived by applying a wavelet and scaling function to an image that has shifted and scaled pixel values^40^. The wavelet decomposition across the whole sMRI image domain at scale (s) and location $(l_1,l_2)$ is defined as follows:

**Fig. 3:**
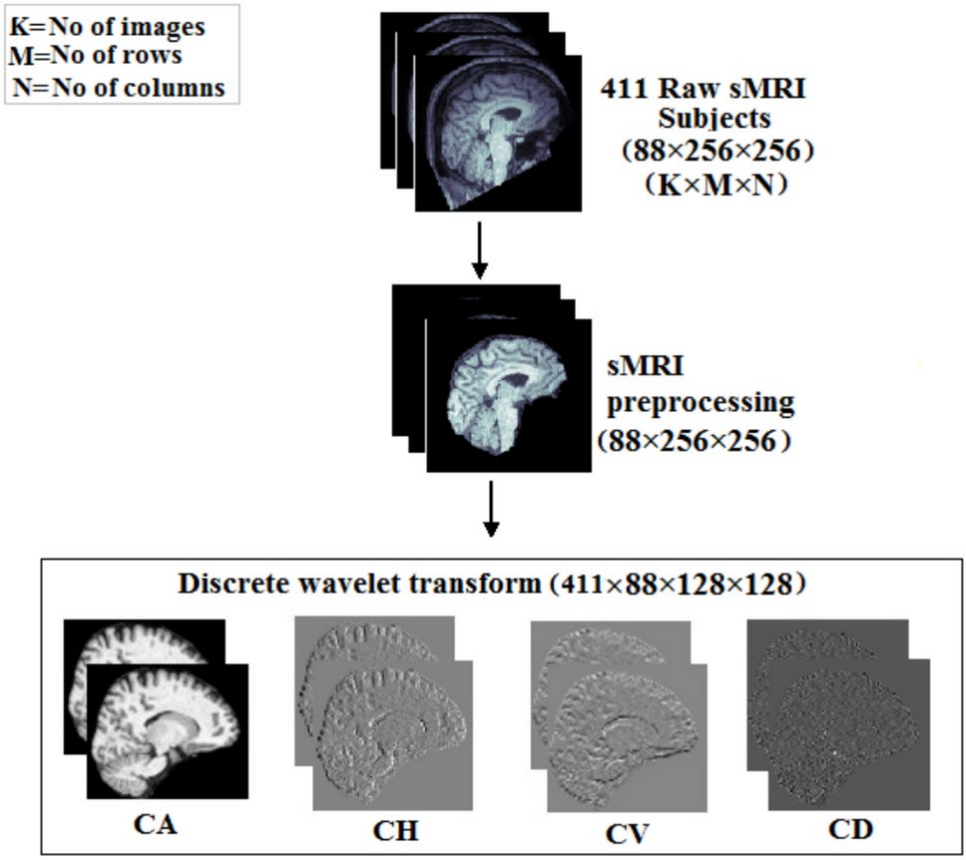
DWT decomposition of sMRI.

The approximation coefficients (*CA*):

1 $$\begin{aligned} \text {CA}_i^j(u,v)=CA[s,l_1,l_2]= \sum _{x,y} I_i^j(x,y) \cdot \Phi \left( \frac{x-2^s l_1}{2^s}, \frac{y-2^s l_2}{2^s}\right) \end{aligned}$$

Horizontal detail coefficients (*CH*):

2 $$\begin{aligned} \text {CH}_i^j(u,v)=CH[s,l_1,l_2]= \sum _{x,y} I_i^j(x,y) \cdot \psi _H \left( \frac{x-2^s l_1}{2^s}, \frac{y-2^s l_2}{2^s}\right) \end{aligned}$$

Vertical detail coefficients (*CV*):

3 $$\begin{aligned} \text {CV}_i^j(u,v)=CV[s,l_1,l_2]= \sum _{x,y} I_i^j(x,y) \cdot \psi _V \left( \frac{x-2^s l_1}{2^s}, \frac{y-2^s l_2}{2^s}\right) \end{aligned}$$

Diagonal detail coefficients (*CD*):

4 $$\begin{aligned} \text {CD}_i^j(u,v)=CD[s,l_1,l_2]= \sum _{x,y} I_i^j(x,y) \cdot \psi _D \left( \frac{x-2^s l_1}{2^s}, \frac{y-2^s l_2}{2^s}\right) \end{aligned}$$

Where,

- $CA[s,l_1,l_2]$ represents the approximation coefficients at scale *s* and locations (*l*1, *l*2).
- $CH[s,l_1,l_2]$, $CD[s,l_1,l_2]$, $CV[s,l_1,l_2]$ represent the horizontal, diagonal, and vertical detail coefficients at scale *s* and locations $(l_1,l_2)$.
- $I_i^j(x,y)$ is the pixel value of the image for subject $i$, image $j$, at coordinates $(x,y)$.
- $\Phi$ is the scaling function for approximation.
- $\psi _H$, $\psi _D$, and $\psi _V$ are wavelet functions for horizontal, diagonal, and vertical details, respectively.

The procedure is applying wavelet decomposition, as illustrated in Fig. 3, to the brain sMRI^12,26,27^. The type of wavelet to be used in the transformation process is ’db1’, also known as the Daubechies wavelet with one vanishing moment^26^. Daubechies wavelets are a family of orthogonal wavelets known for their compact support^27^. To extract relevant characteristics from the sMRI^41^, we use the first-level DWT. This decomposition enables us to capture both coarse-level information represented by approximation coefficients and fine-grained details included in detail coefficients. This characteristic makes it ideal for medical image processing jobs requiring boundary delineation or the identification of subtle structural differences. Therefore, the approximation coefficients and detail coefficients together compress each sMRI image. These coefficients not only compress the visual information but also capture the important structural details while filtering out the high-frequency noise. This compressed representation makes it easier to analyze and extract features later in the data processing pipeline. Further, it enables effective storage and retrieval of relevant information that adds up to improved diagnostic and predictive skills in medical image applications. Biomarkers relevant for schizophrenia may be formed by the wavelet subbands resulting from sMRI^42^.

### Analysis of transform techniques and frequencies performances for image processing

**Fig. 4:**
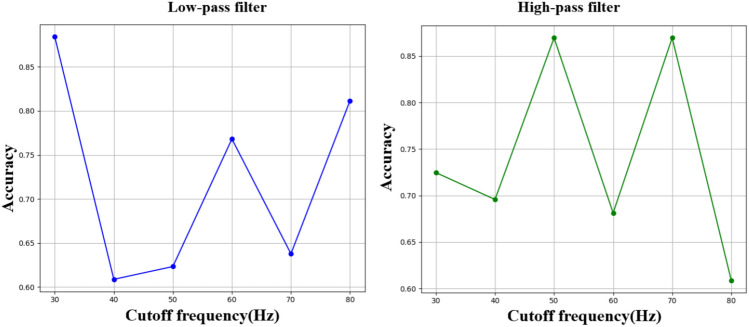
Ablation experiment on low and high frequency elements of sMRI image.

The Daubechies db1 wavelet is chosen due to its unique balance between temporal and frequency localization^46^. Wavelets are useful for image denoising and segmentation because of their ability to study images at different scales^47^. Wavelets outperform typical Fourier-transform-based imaging, owing to their unique ability to localize in both the temporal and frequency domains^48^. They can successfully isolate and decrease noise at higher frequencies, as well as identify and discriminate features with varying resolutions. This multiresolution approach helps to maintain crucial details while reducing extraneous information, which is especially significant in medical imaging where precision and clarity are critical^47,48^. Compared to other transforms, such as Fourier Transform (FT), Hilbert Transform (HT), and Hilbert-Huang Transform (HHT), DWT is better suited for analyzing sMRI images. FT, HT, and HHT are more appropriate for analyzing magnitude and phase. The performance comparison the above mentioned transforms is illustrated in Table 3, as they focus on frequency elements or instantaneous frequencies^45,49,50^. To explore the impact of frequency elements on model efficiency for SZ classification, an ablation study is performed using low-pass (LPF) and high-pass (HPF) filters at different cutoff frequencies. The goal is to examine the performance differences when keeping or eliminating certain frequency ranges. Cutoff frequencies are used at 10 Hz intervals, spanning from 30 Hz to 80 Hz. The observed performance values show an initial performance with a training accuracy of 88.41% at 30 Hz. Nevertheless, as the cutoff frequency rises, performance decreases notably to 60.87% at 40 Hz and stays quite low until it improves to 81.16% at 80 Hz. This indicates that low-frequency components hold essential structural information vital for model efficacy, while adding high frequencies at first diminishes performance, possibly because of noise from less pertinent high-frequency elements. The HPF outcomes show a different trend. The accuracy starts at 72.46% with a cutoff of 30 Hz and diminishes slightly at 40 Hz. The performance reaches a maximum of 86.96% for cutoff frequencies of 50 Hz and 70 Hz, suggesting that mid-frequency elements play a crucial role in the classification of SZ. Outside this range, accuracy drops back to 60.87% at 80 Hz. The ablation study shown in the Fig. 4 that low-frequency components are essential for model effectiveness, demonstrated by the good initial performance observed with LPF set at 30 Hz. When separated using a high-pass filter, high-frequency components exhibit some significance in the range of 50-70 Hz, but typically diminish performance at more extreme cutoff frequencies due to the absence of details. The results indicate that low- and mid-frequency components hold significant information. Therefore, choosing frequency bands can improve the model efficiency in SZ classification.

**Table 3:** Comparison of DWT, Fourier, Hilbert, and HHT transform.

Transform	Purpose in image processing	Output	Advantages	Limitations	Applications
Wavelet transform^42^	Multi-scale decomposition	Approximate + Detail Coefficients (cA, cH, cV, cD)	Good for non-stationary signals (EEG, MRI), noise removal. Downsampling reduces the amount of data	Computationally expensive for high-resolution images	Useful for image compression, denoising, and edge detection
Fourier transform^43^	Converts spatial domain into frequency components	Magnitude and Phase Spectra	Excellent for frequency domain analysis. Good for periodic signals (EEG, fMRI)	Loses time information (only shows global frequency). Phase information is difficult to interpret in images	Used for filtering, pattern recognition, and image enhancement
Hilbert transform^44^	Extracts phase and envelope information	Analytic Signal (Magnitude, Phase)	Useful for examining images that are dynamic or change over time such as satellite imagery or video frames.	Does not provide frequency decomposition (unlike FT and WT). Sensitive to noise (artifacts may appear in the envelope)	Useful in edge detection and phase-based image analysis
Hilbert-Huang transform^45^	Decomposes images into adaptive frequency components	Intrinsic Mode Functions + Hilbert Spectrum	Adaptive and data-driven. Good for highly non-stationary and nonlinear signals	Computationally expensive. IMFs may suffer from mode mixing (false components)	Useful for texture analysis

### ROI features from sMRI

The regions from both L and R hemispheres are described in Table 4. Region Of Interest (ROI) features include the temporal cortex, parietal cortex, precentral cortex, insula, lateral ventricle, prefrontal cortex, putamen, hippocampus, amygdala, thalamus, caudate, cingulate cortex both anterior and posterior parts, and the third and fourth ventricles. It can be seen from the outcome of the analysis that energy values in these regions are lower in the sample from individuals with schizophrenia compared to healthy controls. Research findings align with the general understanding that schizophrenia entails widespread disruptions in the structural and functional integrity of the brain. For instance, the parietal cortex and temporal cortex have been found to be affected in terms of volume and activity in schizophrenia; such regions are important for higher cognitive functions that include decision-making, memory, and emotional regulation^51,52^. Structural defects in schizophrenia include the hippocampus^51,52^, which is responsible for memory and spatial orientation, and the amygdala^53^, which is implicated in emotional processing; these are often found to be structurally smaller in subjects with schizophrenia. The precentral cortex is responsible for voluntary motor control, Dysfunction in the precentral cortex may get involved in these motor symptoms, but it is more commonly associated with sensory and cognitive abnormalities^54^. Similarly, alterations in the insula involved in interoception and emotional processing^55^, and in the cingulate cortex, a critical area for emotion and cognitive control, are frequently found in this disorder^55^ . Furthermore, basal ganglia structures, such as putamen^55,56^, Thalamus^57,58^ and Caudate^59^ are involved in motor control and reward processing. Impairment of these structures can explain features such as abnormal motor activity and cognitive impairments in schizophrenia. Finally, the changes observed in the structures of the ventriculars, such as the lateral ventricles and the third and fourth ventricles^56,57^,agree with a long-established observation of ventricular enlargement in schizophrenia and, therefore, can reflect neurodegeneration or loss of brain tissue. In general, the decline in energy in these regions reflect neurobiological alterations underlying schizophrenia that include both gray-white matter loss as well as disruption to the connectivity and function of such brain areas. These findings contribute to the growing body of evidence that implicates particular brain regions in the pathophysiology of schizophrenia and provide further support to the concept that schizophrenia is a disorder of wide-ranging brain abnormalities in both structure and function.

**Table 4:** ROI features in 88 images from sMRI.

S.no.	ROI feature	Slie number
1	Temporal Cortex_L	11
2	Parietal Cortex_L	23
3	Precentral_L	25
4	Insula_L	31
5	Lateral_Ventricle_L, Prefrontal Cortex_L	40
6	Putamen_L	44
7	Hippocampus_L	50
8	Amygdala_L	52
9	Thalamus_L , Caudate_L	56
10	Cingulate_Ant_L	58
11	Third_Ventricle , Fourth_Ventricle	60
12	Cingulate_Ant_R	62
13	Thalamus_R ,Caudate_R	65
14	Amygdala_R	66
15	Hippocampus_R	68
16	Putamen_R	70
17	Lateral_Ventricle_R, Region Prefrontal Cortex_R	74
18	Insula_R	78
19	Precentral_R	80
20	Parietal Cortex_R	84
21	Temporal Cortex_R	86

## Proposed method and performance analysis

The sMRI scans are stacked together and presented in .nii file format for each subject. A certain number of initial and final images do not contain enough details; hence those images are omitted from further processing. The MDCNNs are trained by the DWT decomposed subbands for which the subband selection is carried out using a certain strategy. The following section elaborates on the subband selection for each CNN.

### Code availability

The Python code with a detailed description for this study is available at https://doi.org/10.5281/zenodo.14994644. The open dataset links are also provided in the repository. Further details and instructions for use are provided in the repository’s README file.

### Wavelet and subband selection for MDCNNs

In this study, the analysis of sMRI data using wavelet transform provides the necessary spatial and frequency localization for effective feature extraction. Another important reason for DWT is subsampling. It reduces the amount of data by half and subsequent computations in the layers of multidimensional CNN. The capacity of the Daubechies db1 wavelet to retain crucial image properties while suppressing the noise improves the model’s accuracy. As seen in Table 5, Daubechies db1 consistently performs good for all CNN architectures. Daubechies db1 wavelet delivers improved accuracy in comparison to other wavelets such as Daubechies db2, Symlets, and Coiflets. The proposed model presents a significant advance in integrating DWT and CNN. The proposed model not only includes wavelet coefficients-based feature extraction, but also ensembles multidimensional classification with the specific wavelet features. The selection of CD, CH, and CV subbands is not arbitrary. Each sub-band is chosen based on its ability to capture key anatomical, spatial, and volumetric elements of the brain impacted by schizophrenia. The experimental findings, which include ablation trials presented in Table 5 lend more support to the selection of different subbands. The CD sub-band excels at gathering minute structural details, the CH sub-band focuses on spatial patterns, and the CV sub-band is good at recognizing volumetric elements. These subbands work together to form a comprehensive and successful categorization model for schizophrenia prediction. The feature extraction strategy also proves vital in the selection of subbands. 1DCNN deals with energy features of subands attained an accuracy of 99.75%. The sum of gradient magnitude of CD coefficients presents a better landscape revealing the abnormalities in the brain tissues. The coefficients in CH subband prove to be effective for exploiting spatial correlation of SZ features in 2D space. In 2D-CNN, the model training and classification is entirely based on 2D-feature maps at an accuracy of 99.51%. The experimental study proves that the 2D feature maps extracted from the CH subband better reveals the anatomical and functional changes in the brain tissues. In 3D-CNN, the feature extraction is carried out in spatial-temporal space using 3D kernels . The extracted features from the CV subband better representing the spatial-temporal characteristics of brain tissues pertaining to the intended task at an accuracy of 99.69%. Overall, applying DWT to sMRI images and then using each subband for classification improves accuracy compared to the spatial sMRI data. This suggests that DWT subbands deliver better classification while also providing a resource-efficient solution. It is observed that the classification in the DWT domain improves the performance of classification using spatial sMRI data.

**Table 5:** Illustration of wavelet and subband selection for MDCNNs.

Wavelet transform	1DCNN	2DCNN	3DCNN
**sMRI (without DWT)**			
**sMRI**	91.98	90.09	80.66
**Daubechies (db1)**			
CA	98.54	98.17	99.13
CH	98.17	99.51	98.78
CV	88.30	97.86	99.69
CD	99.75	98.54	98.51
**Daubechies (db2)**			
CA	83.53	85.67	86.78
CH	85.45	87.54	78.88
CV	76.44	79.12	88.29
CD	90.54	89.76	87.65
**Symlets (sym2)**			
CA	84.12	87.34	88.22
CH	93.25	94.05	78.56
CV	88.89	87.22	90.98
CD	98.39	97.45	96.78
**Coiflets (coif1)**			
CA	85.43	84.78	86.89
CH	78.94	82.95	80.12
CV	78.66	79.45	81.25
CD	95.97	84.44	83.17

**Fig. 5:**
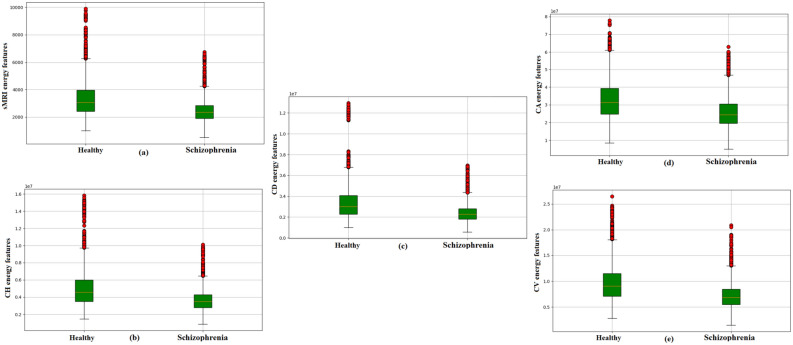
Energy distribution of healthy control and schizophrenia images. (**a**) sMRI in the spatial domain (**b**) CH subband (**c**) CD subband (**d**) CA subband and (e) CV subband.

There are 88 images chosen from each subject from the input sMRI file format. The proposed method involves feature extraction with the help of wavelet coefficients from sMRI. The extracted features from the DWT domain are fed to MDCNN for classification. The energy values evaluated from CD subbands of 88 images will contribute to a one dimensional array of size $1\times 88$ for each subject. Therefore, a 1D CNN can capture the high frequency patterns from this one-dimensional data. This subband is helpful in characterizing the texture of the brain and in finding structural problems in the subcortical regions. Texture analysis can determine the changes in tissue organization and heterogeneity, which are associated with neurodevelopmental problems in schizophrenia. Figure 5 shows a boxplot of all four DWT subbands and the sMRI data comparing their energy characteristics. This graphical illustration shows that the energy-related properties of the CD subband are very different in both healthy and schizophrenic cases in comparison to other subbands. Thus, the gradient magnitude as energy feature in the CD subband makes it essential in discriminating between the two classes while improving the classification accuracy related to schizophrenia diagnosis. The CD subband is sensitive to alterations in local edges, which correlate to tiny structural features critical for diagnosing schizophrenia-related disorders such as brain tissue loss and surface area changes.

**Fig. 6:**
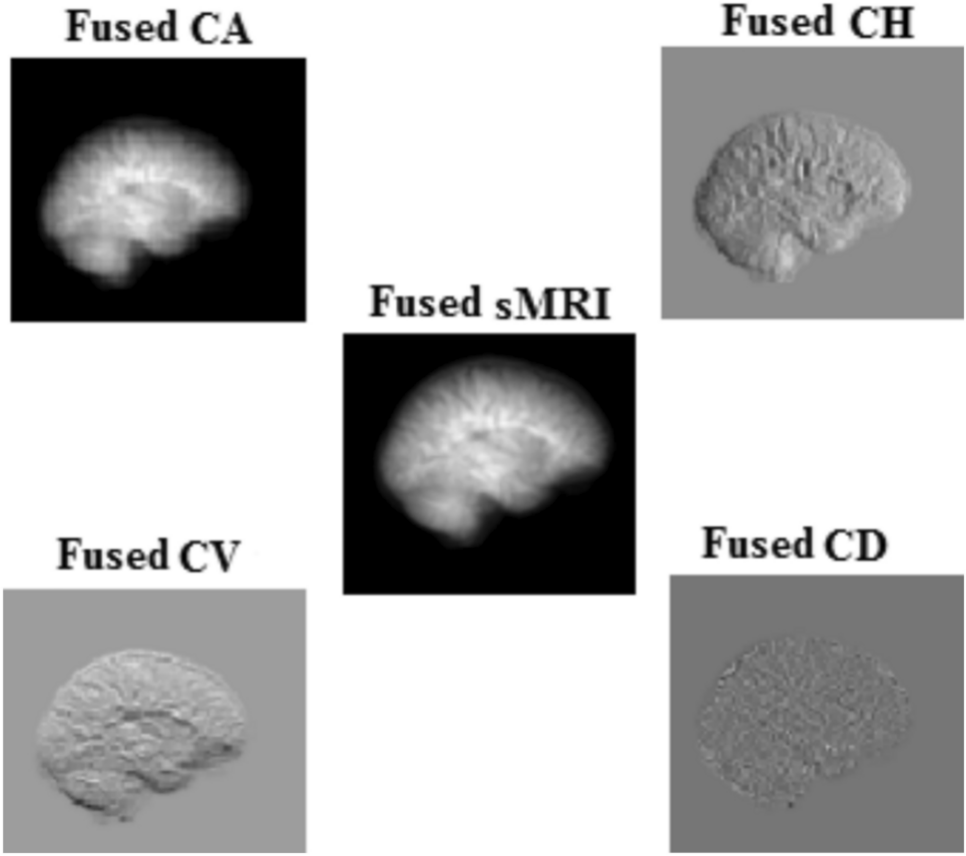
DWT decomposition of sMRI for 2DCNN.

CH subband tends to express the abnormalities of schizophrenia in the horizontal pattern. Hence, a 2D CNN is applied to extract the spatial relationship in the two-dimensional DWT space. The 2D-CNN is trained and validated by the DWT coefficients and sMRI data separately to analyze the performance in the DWT domain. Figure 6 illustrates the fused sMRI for a subject and the decomposed DWT subbands. Among the four subbands, the CH subband outperforms the others in classification. Spatial features collected from the CH sub-band are fed into the 2D-CNN model, which is ideal for analyzing the spatial attributes of the brain’s cortical structures. The 2D-CNN uses spatial coherence with respect to horizontal frequency features to successfully capture the morphological alterations associated with schizophrenia.

The 3D CNN focuses on the volumetric relationship between healthy and schizophrenia feature maps, such as vertical abnormalities from the regions of insula and corpus callosum, which are critical for schizophrenia classification. It also extracts spatial-temporal characteristics in relation to frequency alterations in the CV subband. The stacked images in sMRI and its four DWT subbands, as shown in Fig. 3, are used as volumetric data for classification in the 3D-CNN model. Lastly, the multidimensional CNN models are applied to different DWT subbands will make it possible to improve model capabilities on detection and further analysis in the areas of abnormalities.

### Schizophrenia classification using 1DCNN from CD subband

**Fig. 7:**
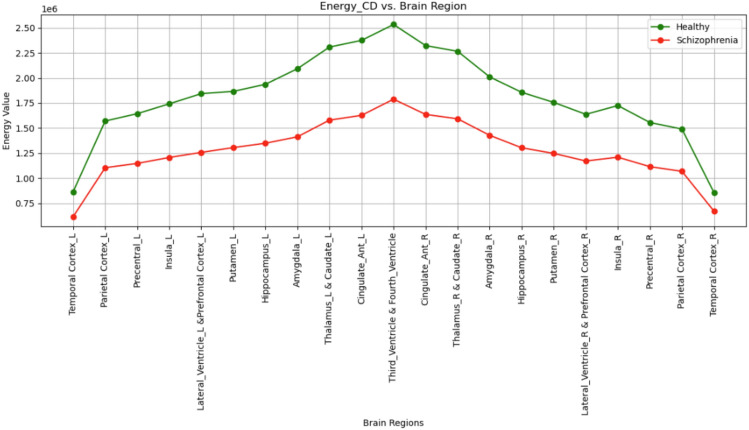
Energy distribution of healthy control and schizophrenia images.

**Fig. 8:**
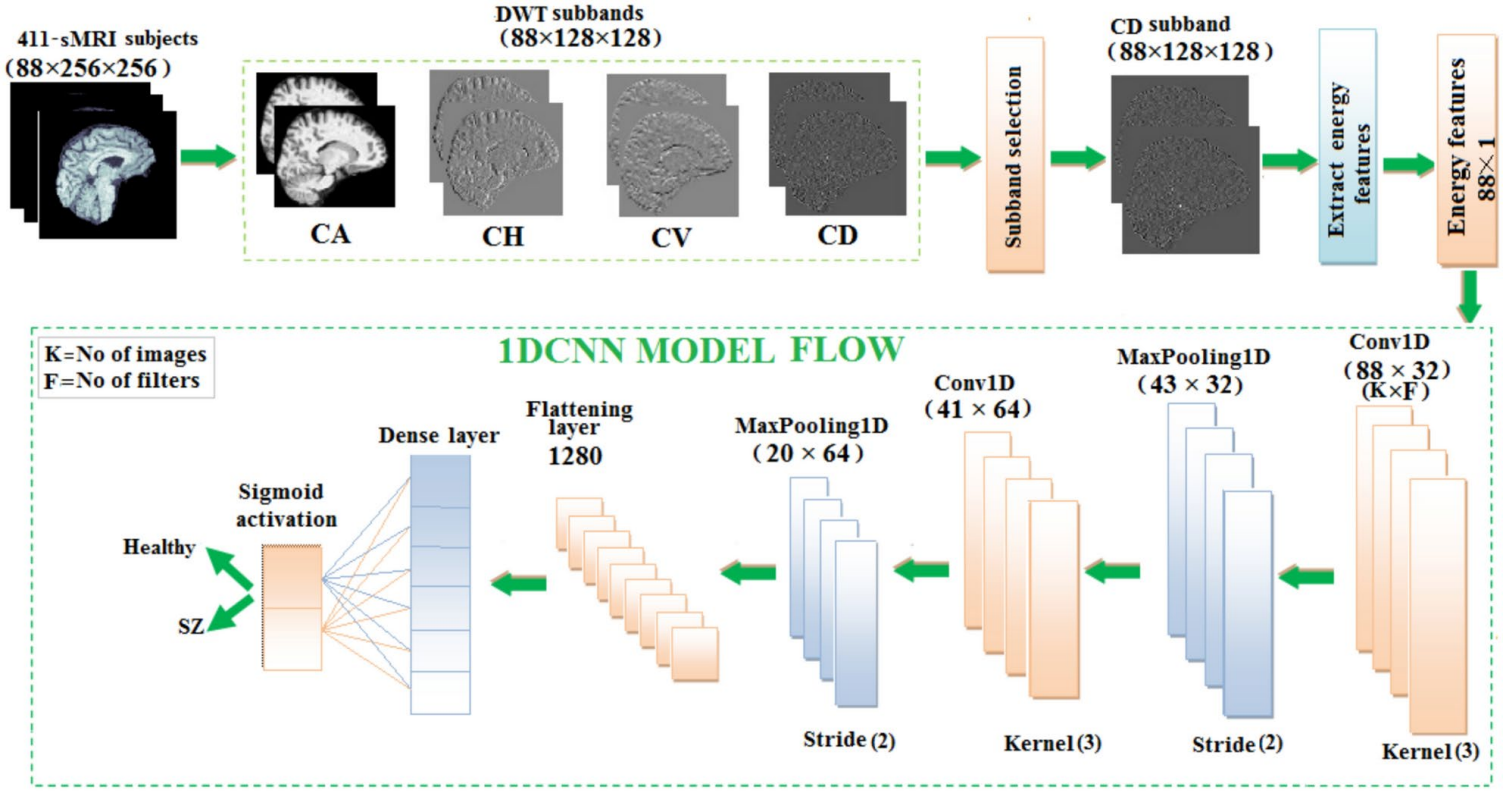
Flow diagram of 1DCNN for schizophrenia classification.

The 1D-CNN model for schizophrenia classification makes use of CD subband decomposed from the DWT. The sMRI data from a subject is organized as 88$\times$ 256$\times$256 images, which represent three-dimensional medical volumes. After DWT decomposition, the coefficients become 88$\times$ 128$\times$128. The goal is to extract energy characteristics from the CD subband for training and testing the 1D-CNN model. This study indicates that the energy values exhibit better difference between schizophrenia and healthy subjects as shown in Fig. 7. The distinction in energy values proves to be the valuable information for classification. From a set of 88 images, energy values representing 26 features in the specific brain region were evaluated to differentiate the individuals with schizophrenia to healthy controls. Though other coefficients can capture structural features as well, the CD coefficient is performs better in observing the structural changes typical in schizophrenia, especially to brain curvature and subtle abnormalities in shape. The gradients are a measure of structural intensity or variation in the brain tissue characteristics. The gradient magnitude essentially measures how much the intensity of the tissue changes between neighboring pixels in the MRI image, capturing textural patterns, edges and fine details. Generally, higher magnitudes of gradient correspond to areas of higher contrast, and the lower magnitudes correspond to smoother or less contrasted regions. The energy values, both from the sMRI data and wavelet coefficients, are generally lower in schizophrenia than in healthy individuals. This may suggest that the intensity variation, contrast, finer details, and gradients in brain structures of the schizophrenia group are lower compared to those in the healthy group. The anomalies in structural organization that are due to reduced cortical volume, changes in grey matter, or altered brain texture are evident in the schizophrenia diagnosis. After computing the energy features for each image in a single subject’s three-dimensional structure, which yields (1$\times$88) energy values per subject, the features are fed into a 1D-CNN model. 1D-CNN model architecture illustrated in Fig. 8, starts by examining the input data with a 1D convolution layer that uses ReLU activation to detect key patterns. It uses a max-pooling layer to highlight important features while reducing the data to reduce the complexity. This process is repeated with another 1D convolution layer, followed by max-pooling to refine the retrieved patterns even further. Finally, the model generates a prediction using a dense output layer with a single neuron and sigmoid activation, evaluating the processed information via a decision-making stage that calculates the likelihood of a specific outcome, allowing for accurate input data classification. This 1D-CNN architecture efficiently detects temporal patterns in sequential data, relying on extracted energy features to achieve high accuracy in medical image classification. The 1D-CNN’s capacity as presented in Table 6 to detect the patterns in sequential data successfully uses the extracted energy features to classify with high accuracy.

**Algorithm 2:**
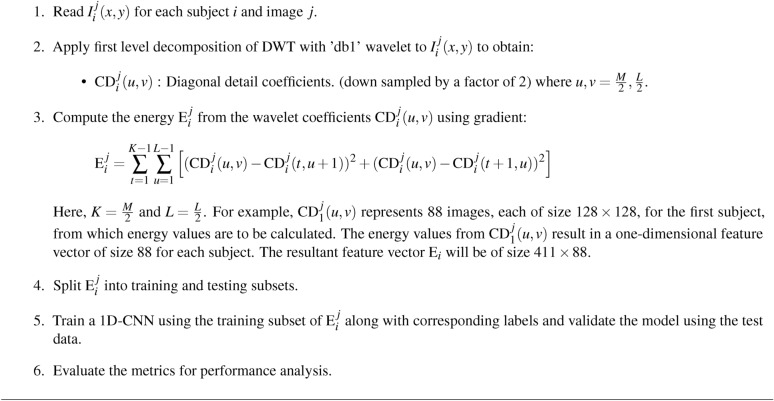
Classification using 1D-CNN

**Table 6:** Conv1D Model parameters.

Layer type	Kernel size	Output shape
Conv1D	(3$\times$32)	(86, 32)
MaxPooling1D	(2)	(43, 32)
Conv1D	(3$\times$64)	(41, 64)
MaxPooling1D	(2)	(20, 64)
Flatten	–	(1280)
Dense	(64 units)	(64)
Dense	(1 unit)	(1)
Total parameters		**88,385**
Trainable parameters		**88,385**
Non-trainable parameters		**0**
Learning rate		0.001
Activation functions		ReLU (hidden layers), Softmax (output layer)
Optimizer		Adam
K-Fold Cross Validation	Number of folds	5
Epochs		20
Batch size		32
Callbacks		TensorBoard, ModelCheckpoint

**Table 7:** Performance analysis of 1D-CNN for all the subbands.

Metrics (%)	DWT subbands			
	CA	CH	CV	CD
Accuracy	98.54	98.37	98.17	**99.75**
precision	99.38	97.77	99.24	**100**
sensitivity	97.75	99.28	96.35	**99.43**
specificity	99.17	96.35	99.45	**100**
F1-score	98.76	99.47	97.77	**99.71**

**Fig. 9:**
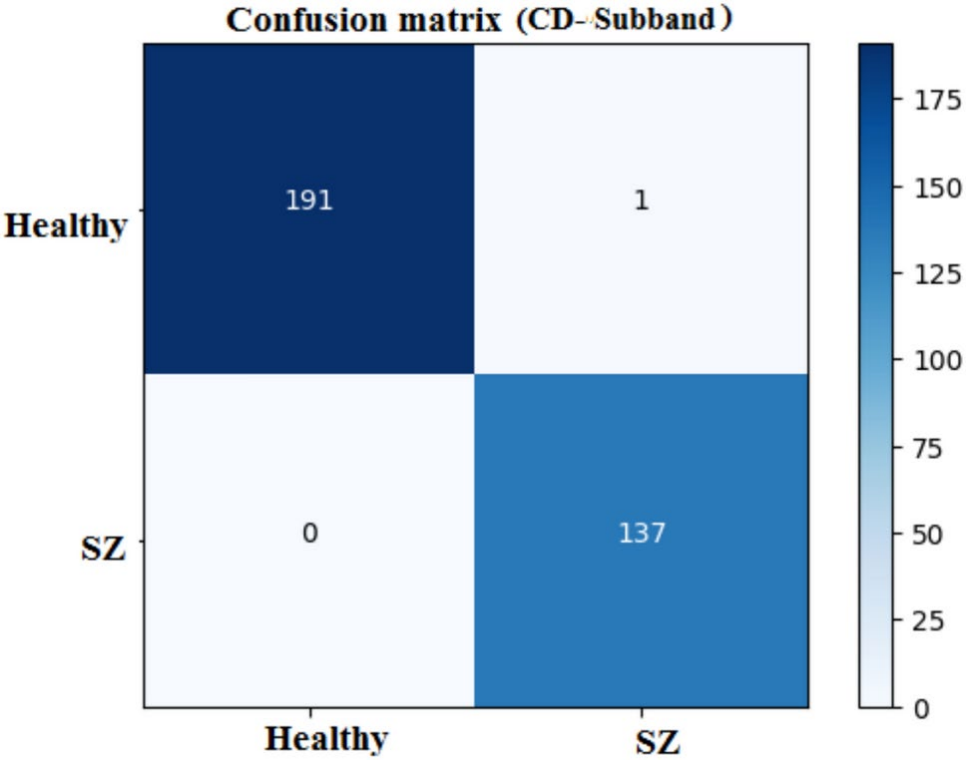
Confusion matrix of 1D-CNN.

Table 7 depicts the performance metrics for the 1D-CNN using four DWT subbands. In all the subbands, CD has very high precision and accuracy, thus reliable to detect actual positive and negative cases. Precision in all the subbands was always very high, showing that the model could always identify the presence of a positive case. Sensitivity scores represent the model’s capacity to recognize positive cases, with the CD subband again demonstrating the highest reliability. Specificity is also high, notably for the CD subband, demonstrating that the model can properly detect negative cases. Overall, the 1D-CNN performs well for all the subbands, with notable performance for the CD subband. The balance between precision and sensitivity is well-maintained, as demonstrated in the F1-scores for all subbands, with CD leading performance once again. Overall, the 1D-CNN model performs well across all DWT subbands, with the CD subband outperforming the other DWT subbands.

The confusion matrix as shown in Fig. 9, summarizes the 1D-CNN’s performance, in classifying healthy and schizophrenia. The matrix demonstrates that among the 192 genuine healthy cases, 191 were correctly classified with one misclassification. Similarly, all 137 real schizophrenia cases were correctly classified. This yields a high accuracy, suggesting that the 1D-CNN performs well at differentiating healthy and schizophrenia classes. The matrix shows the sensitivity and specificity with near-perfect classification for both classes.

**Algorithm 3:**
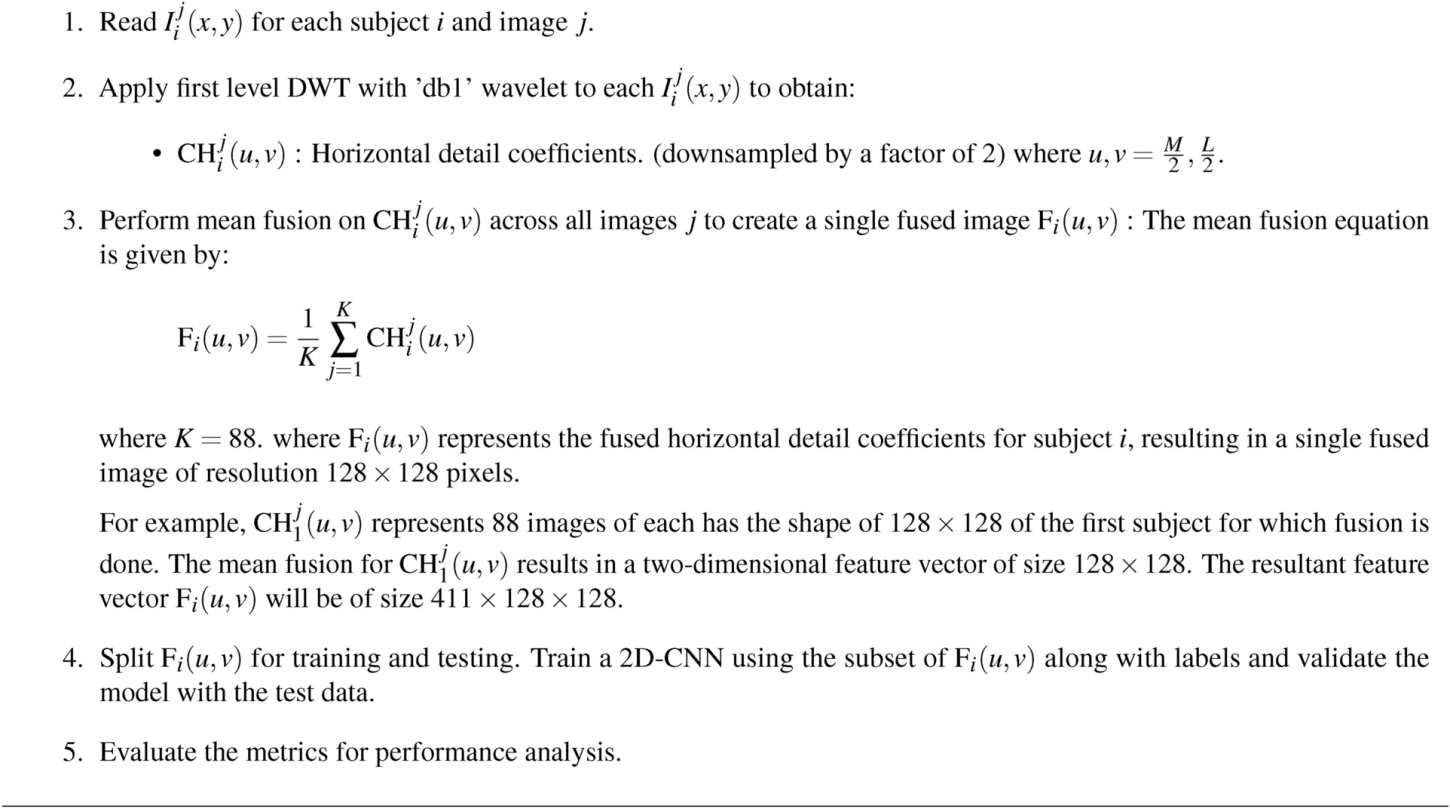
Classification using 2D-CNN

### Schizophrenia classification using 2DCNN from CH subband

**Fig. 10:**
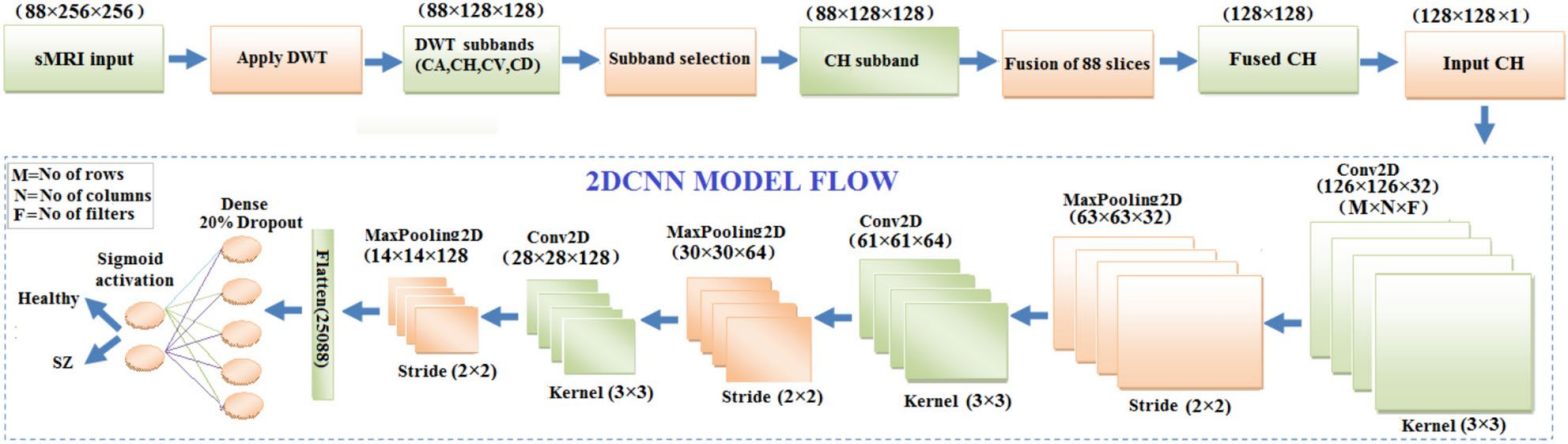
Flow diagram of schizophrenia classification using 2DCNN using CH subband.

**Fig. 11:**
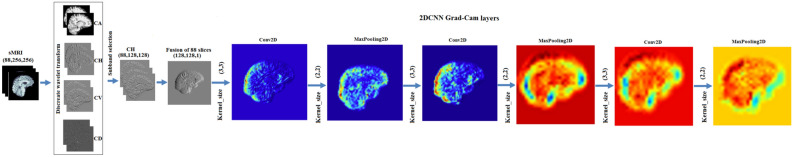
The image contains Grad-CAM features derived from each layer of the 2DCNN neural network. These features capture hierarchical patterns ranging from basic edges and textures to more complex structures, allowing the network to learn discriminative features for classification and detection tasks.

The 2D-CNN model uses the CH subband from DWT decomposition for schizophrenia classification. The sMRI dataset has CH subbands of 411 subjects each with a size of 88$\times$ 128$\times$128. The mean fusion is applied to the 88 images of a subject resulting in a single fused image of size 128$\times$128. The fused image presents the average of information from all 88 images pixel-wise. The resulting fused image of resolution 128$\times$128 is presented as an input to a 2D-CNN model for additional processing and analysis. The 2D-CNN model parameters are presented in Table 8. The model architecture, shown in Fig. 10, shows a multi-layered architecture that fetches the information from input images in order to do binary classification. The deeper layers take on to proceed with refining these properties using filtering, normalization, and pooling, adding more complicated patterns on the primary features. The modified characteristics are then flattened and sent via a fully connected layer to improve the network’s decision-making capabilities, complete with a dropout mechanism to prevent overfitting. Finally, the output layer generates the classification results. The CH subband, which contains the high frequency components in horizontal direction of the DWT decomposition, can help to resolve small cortical anatomical details that may be impaired in brain regions such as the parietal and precentral lobes. Gradient-weighted Class Activation Mapping (Grad-CAM), as shown in Fig. 11, is applied to highlight the 2D-CNN focus areas. In this case the, parietal cortex and precentral lobes brain areas associated with schizophrenia have structural abnormalities that are necessary for the classification task.

**Table 8:** Conv2D Model parameters.

Layer type	Kernel size	Output shape
Conv2D	(3$\times$ 3$\times$32)	(126, 126, 32)
BatchNormalization	–	(126, 126, 32)
MaxPooling2D	(2$\times$ 2)	(63, 63, 32)
Conv2D	(3$\times$ 3$\times$64)	(61, 61, 64)
BatchNormalization	–	(61, 61, 64)
MaxPooling2D	(2$\times$ 2)	(30, 30, 64)
Conv2D	(3$\times$ 3$\times$128)	(28, 28, 128)
BatchNormalization	–	(28, 28, 128)
MaxPooling2D	(2$\times$ 2)	(14, 14, 128)
Flatten	–	(25088)
Dense	(128 units)	(128)
Dropout (20%)	–	(128)
Dense	(1 unit)	(1)
Total parameters		**3,305,089**
Trainable parameters		**3,304,641**
Non-trainable parameters		**448**
Learning rate		0.001
Activation functions		ReLU (hidden layers), Softmax (output layer)
Optimizer		Adam
K-Fold Cross Validation	Number of folds	5
Epochs		20
Batch size		32
Callbacks		TensorBoard, ModelCheckpoint

**Table 9:** Performance metrics of 2D-CNN for all the DWT subbands.

Metrics (%)	DWT subbands			
	CA	CH	CV	CD
Accuracy	99.17	**99.51**	97.36	98.24
precision	95.80	**98.88**	95.89	98.85
sensitivity	100	**100**	99.29	97.74
specificity	96.87	**99.14**	96.79	99.14
F1-score	97.85	**99.43**	97.56	98.29

**Fig. 12:**
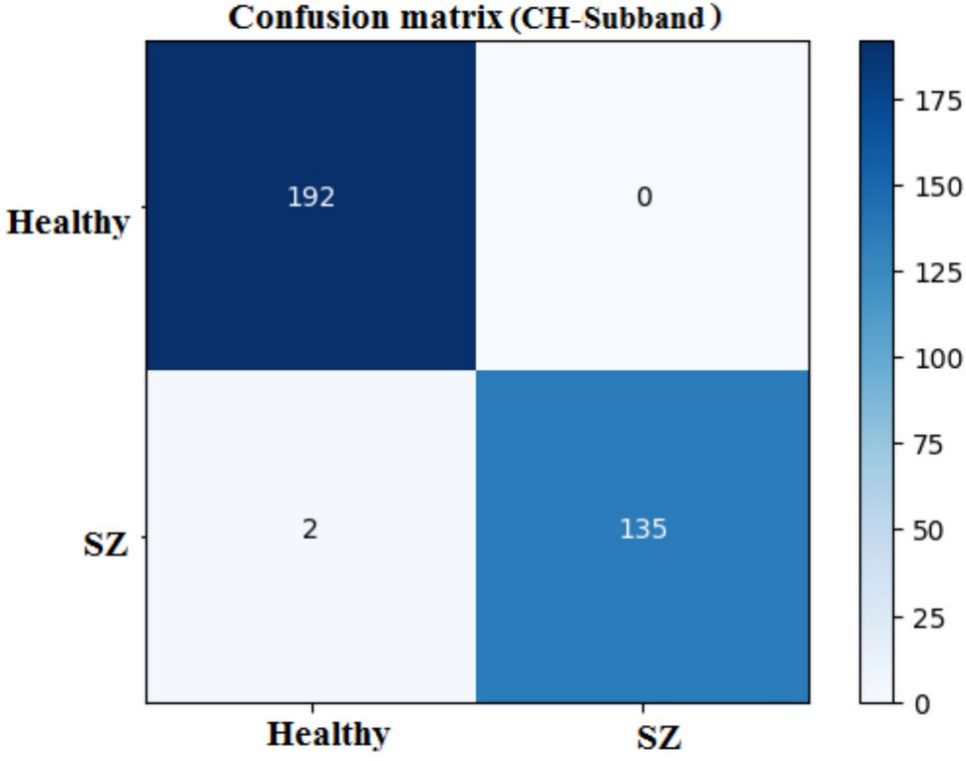
Confusion matrix of 2D-CNN.

Table 9 summarizes the performance metrics of a 2D-CNN based on four subbands. The parameters presented are accuracy, precision, sensitivity, specificity, and F1-score. The 2D-CNN delivers an accuracy of 99.51%, precision of 98.88%, sensitivity of 100%, specificity of 99.14% and F1-score of 99.43%. Accuracy scores continue to be better across all coefficients, showing that the model can classify accurately. Precision, which measures the ability of the model to predict actual positive cases is considerably high, with CH scoring the highest. Sensitivity scores, which represent how the model correctly identifies actual cases. Specificity measures how well the model has actually identified negative cases; for all coefficients, consistency in specificity is seen except in CH, which excels.

The confusion matrix, shown in Fig. 12, reveals that the 2D-CNN properly recognizes 192 healthy cases and 135 schizophrenia patients. However, two healthy patients were misclassified as schizophrenia which is false positive (FP), but no schizophrenia cases were misclassified as healthy which is false negative (FN). This high degree of accuracy represents a low number of misclassifications and demonstrates the efficiency of the spatial characteristics.

**Algorithm 4:**
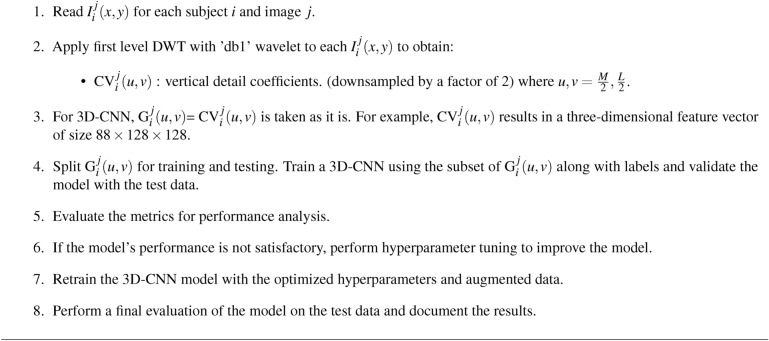
Classification using 3DCNN

### Schizophrenia classification using 3D-CNN from CV subband

**Fig. 13:**
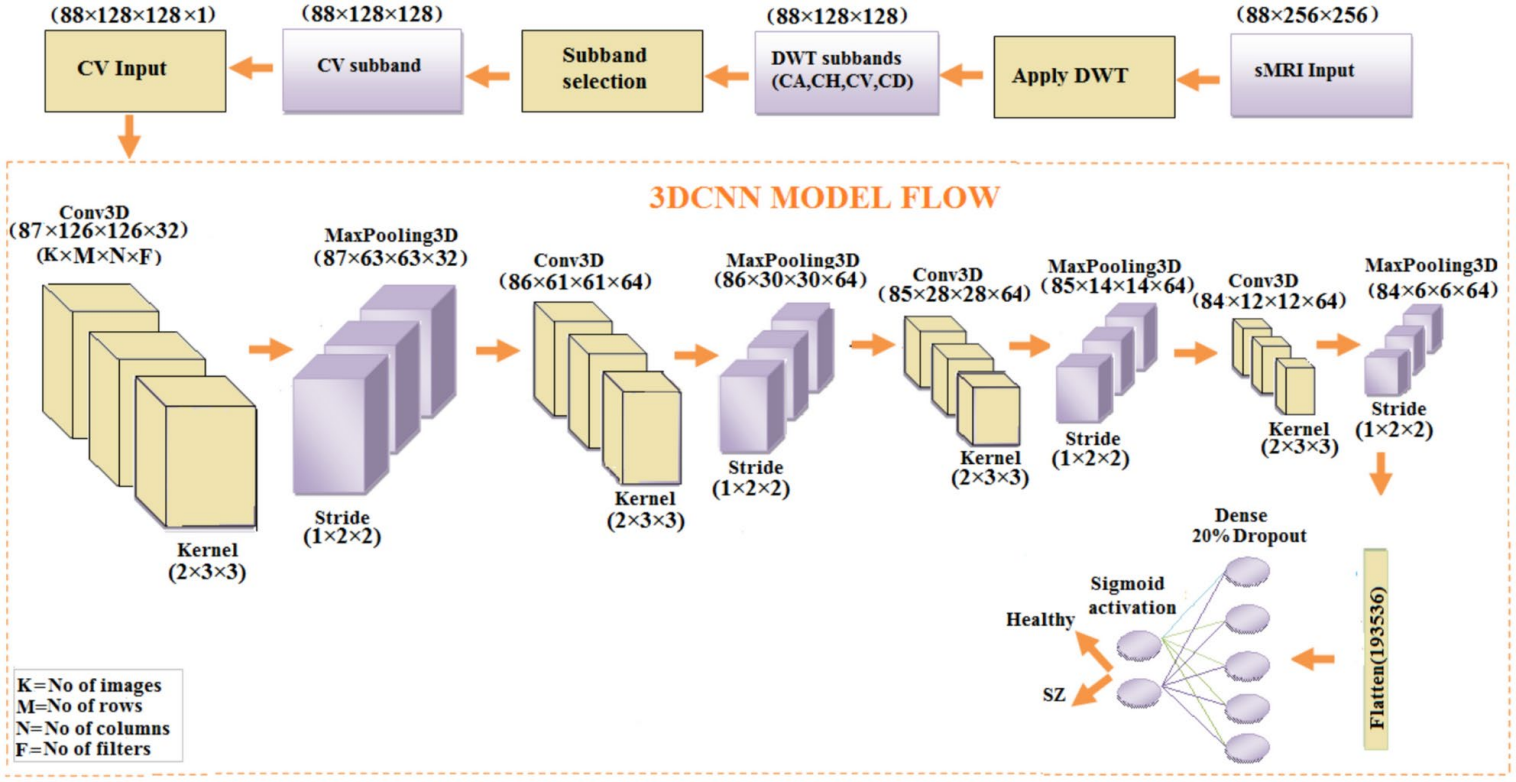
Flow diagram of schizophrenia classification using 3DCNN from CV subband.

**Fig. 14:**
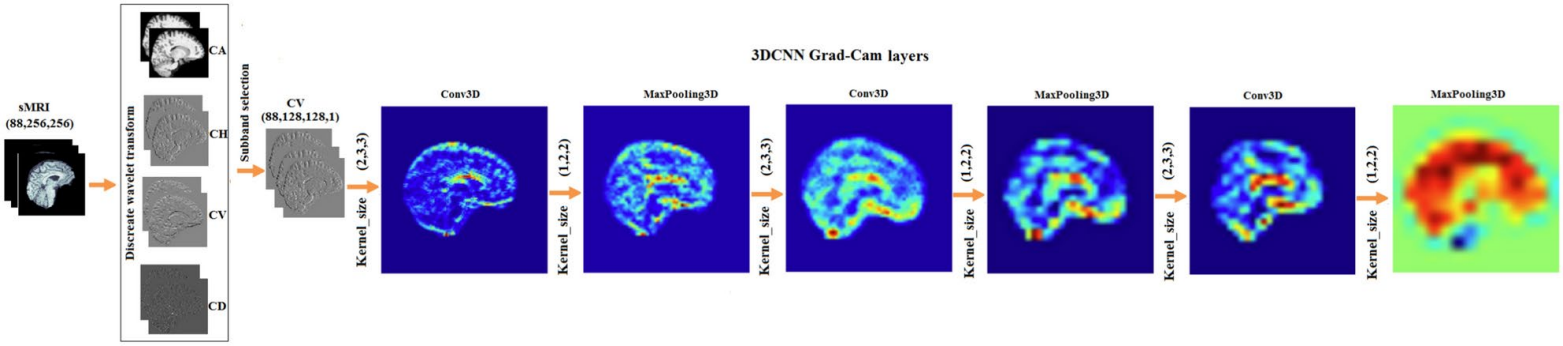
The image shows the Grad-CAM features extracted from each layer in the 3DCNN model, revealing hierarchical representations from finer to more generalized patterns. Each layer’s output contributes to a comprehensive depiction in DWT domain, demonstrating how the network transforms input data into increasingly meaningful representations.

The 3D-CNN model uses the CV coefficients to classify sMRI images. The input data is given as volumetric data of size (88$\times$ 128$\times$128). Input data is decomposed as CV coefficients, which highlight the vertical high frequency information that can be found in MRI scans. This CV subband with 3D-CNN delivers better performance due to the model’s ability to handle volumetric data, allowing the network to capture spatial patterns not just across the 2D dimensions of width and height, but also through the depth of the image, taking into account the surrounding context in the third dimension. The 3D-CNN model flow, shown in Fig. 13, begins with a 3D convolutional layer with 32 filters, utilizing the ReLU activation and anticipating input images of resolution $88\times$ $128\times 128$. Pooling is used along the spatial dimensions to minimize spatial resolution while keeping key features. This method is done three more times with additional convolutional layers, each followed by max-pooling, bringing the total number of filters to 64. This configuration enables the network to gradually extract hierarchical features from the input volumes. Finally, the feature maps are flattened and fed into fully connected layers, beginning with a dense layer of 128 neurons activated by ReLU and progressing to a single neuron with sigmoid activation, which produces the binary classification output. The model architecture comprising layer configurations, parameter counts, network depth, and complexity and the model parameters is presented in Table 10. To improve interpretability, the model employs Grad-CAM to visualize the most influential brain regions that affect decision-making. The highlighted sections, as shown in Fig. 14, are the temporal cortex, parietal cortex, and precentral lobes, corpus callosum which have been shown to be critical for diagnosing schizophrenia.

**Table 10:** Conv3D Model parameters.

Layer type	Kernel size	Output shape
Conv3D	(2$\times$ 3$\times$ 3$\times$32)	(87, 126, 126, 32)
MaxPooling3D	(1$\times$ 2$\times$ 2)	(87, 63, 63, 32)
Conv3D	(2$\times$ 3$\times$ 3$\times$64)	(86, 61, 61, 64)
MaxPooling3D	(1$\times$ 2$\times$2)	(86, 30, 30, 64)
Conv3D	(2$\times$ 3$\times$ 3$\times$64)	(85, 28, 28, 64)
MaxPooling3D	(1$\times$ 2$\times$ 2)	(85, 14, 14, 64)
Conv3D	(2$\times$ 3$\times$ 3$\times$64)	(84, 12, 12, 64)
MaxPooling3D	(1$\times$ 2$\times$ 2)	(84, 6, 6, 64)
Flatten	–	(193536)
Dense	(128 units)	(128)
Dense	(1 unit)	(1)
Total parameters		**24,957,985**
Trainable parameters		**24,957,985**
Non-trainable parameters		**0**
Learning rate		0.001
Activation functions		ReLU (hidden layers), Softmax (output layer)
Optimizer		Adam
K-Fold Cross Validation	Number of folds	5
Epochs		20
Batch size		32
Callbacks		TensorBoard, ModelCheckpoint

**Table 11:** Performance metrics of 3D-CNN for the four DWT subbands.

Metrics (%)	DWT subbands			
	CA	CH	CV	CD
Accuracy	99.13	98.58	**99.69**	98.51
precision	98.18	99.05	**99.27**	97.17
sensitivity	100	97.87	**100**	98.15
specificity	98.38	96.75	**99.47**	99.72
F1-score	99.08	96.77	**99.63**	98.08

**Fig. 15:**
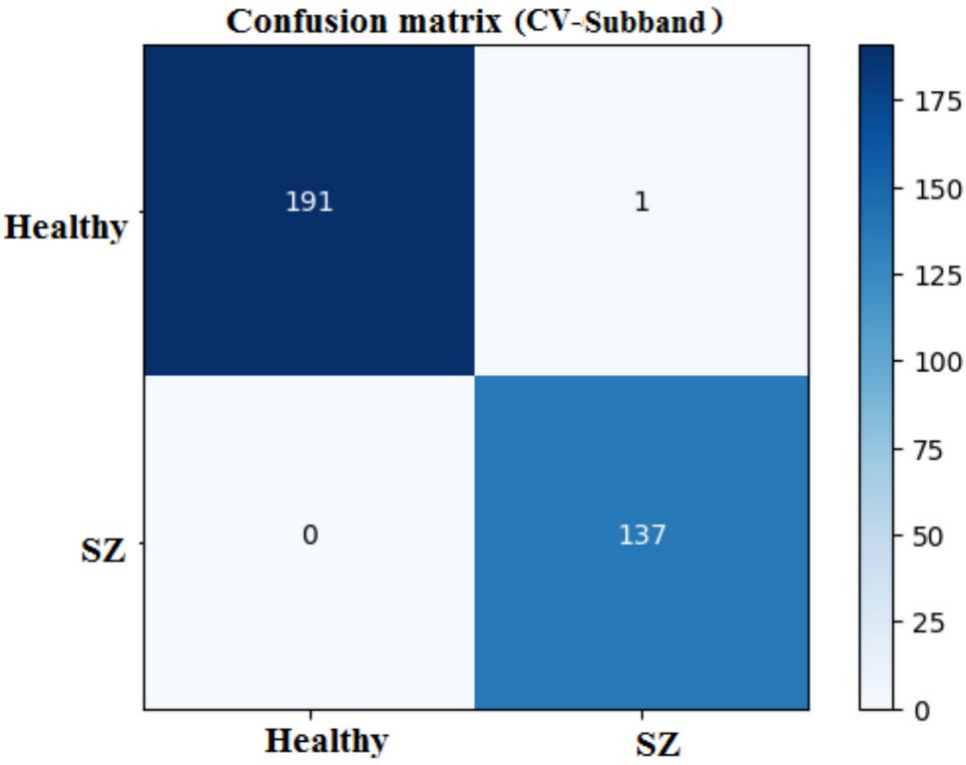
Confusion matrix of 3D-CNN.

Table 11 shows the performance metrics of the 3D-CNN for the four DWT subbands. The CV subband has an accuracy of 99.69%, precision of 98.27%, sensitivity of 100%, specificity of 99.47%, and F1-score of 99.63%. Generally, these metrics have been used to evaluate the four DWT subbands to display the prediction ability of the 3D-CNN model.

The confusion matrix, presented in Fig. 15,shows the performance of the classification model for healthy and schizophrenia classes. The model classifies 191 cases correctly but misclassifies one instance as schizophrenia. The model correctly categorizes all 137 occurrences in the schizophrenia class with no false negatives.

**Algorithm 5:**
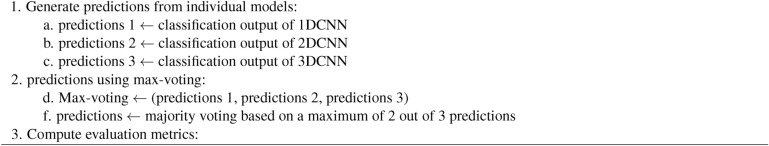
MVMDM

### Confusion matrix and max-voting analysis of MVMDM model

**Fig. 16:**
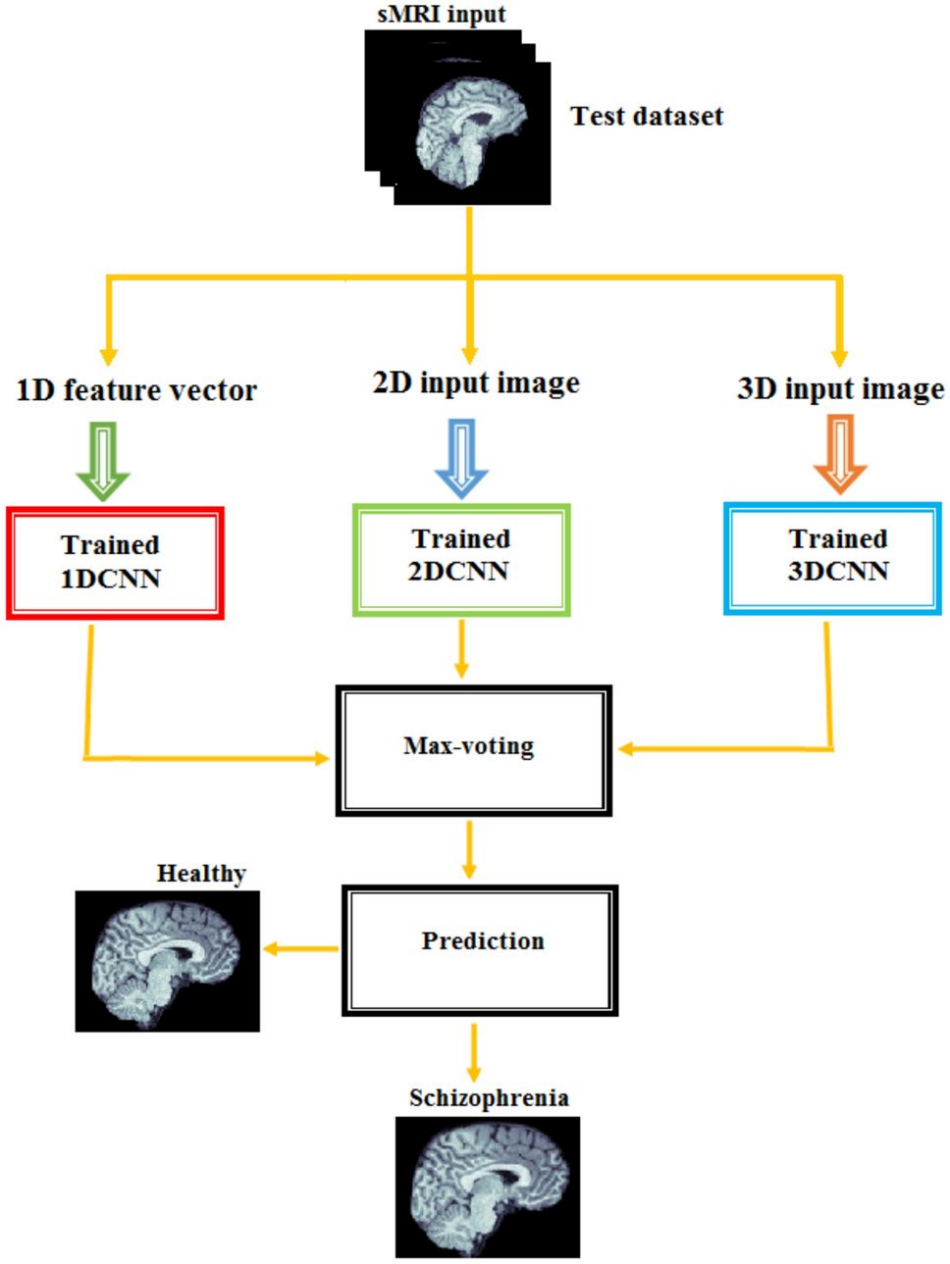
MVMDM model.

The proposed method, depicted in Fig. 16, involves the outputs of 1D-CNN, 2D-CNN, and 3D-CNN models using the max voting ensemble strategy. Initially, the algorithm accepts the prediction of MDCNN models to classify sMRI data into schizophrenia or healthy control. Subsequently, the max voting method is applied to these outputs. Finally, the MVMDM approach returns the projected class based on the results of the max voting ensemble technique. The MVMDM method aims to improve the accuracy of classification tasks distinguishing between individuals with schizophrenia and healthy controls using sMRI data.

**Table 12:** Performance analysis of MDCNNs and MVMDM.

Metrics (%)	Models			
	1D-CNN	2D-CNN	3D-CNN	MVMDM
Accuracy	99.75	99.51	99.69	100
Precision	100	98.88	99.27	100
Sensitivity	99.43	100	100	100
Specificity	100	99.14	99.47	100
F1-score	99.71	99.43	99.63	100

**Fig. 17:**
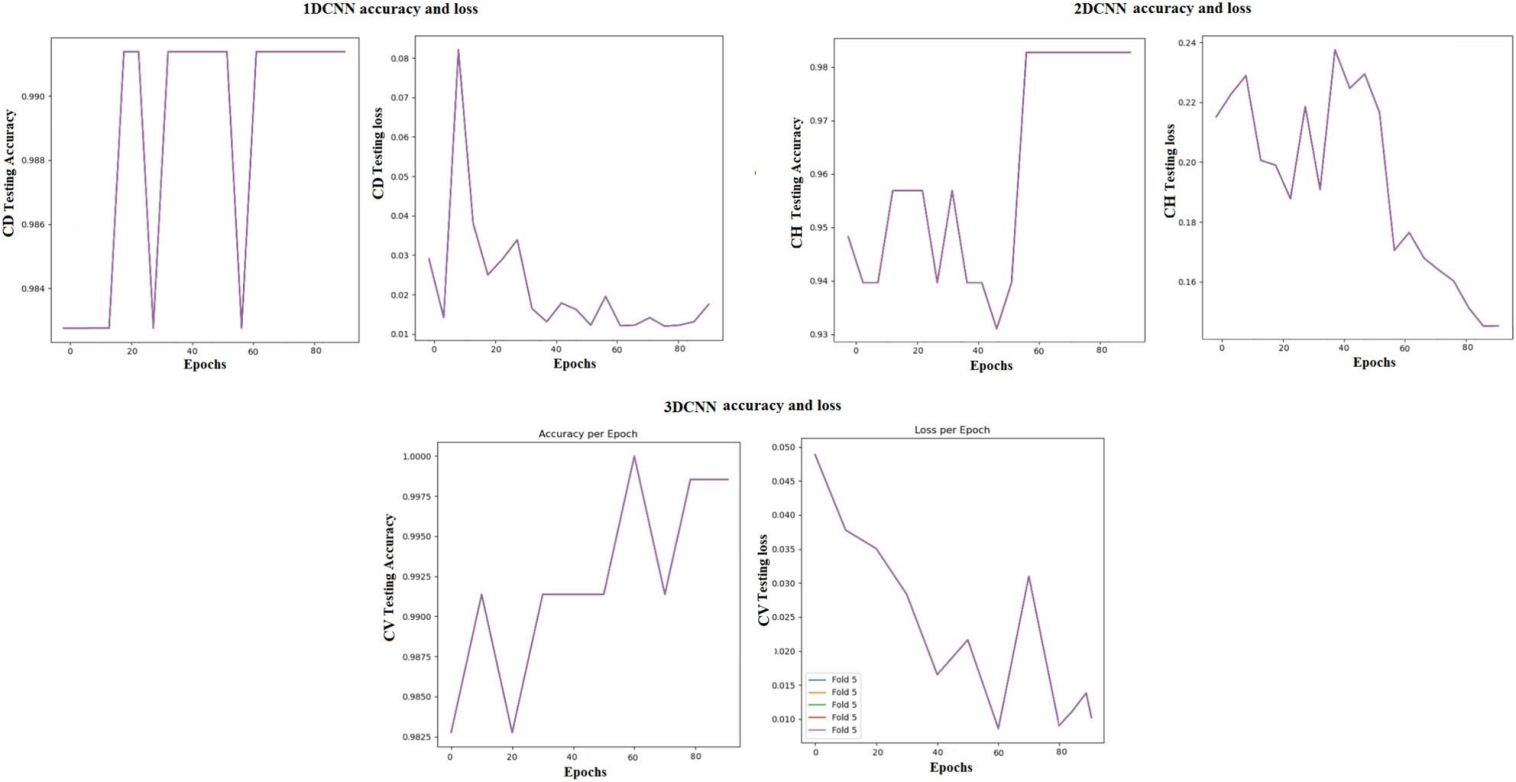
Accuracy and loss graph of MDCNNs model.

Table 12 compares the performance of MDCNNs and the MVMDM model. The MVMDM model, which employs a max-voting strategy to ensemble the output scores of the MDCNNs models, performs well is also represented in accuracy and loss graph as shown in Fig. 17. This max-voting technique enables MVMDM to surpass any individual CNN model, demonstrating its effectiveness in classification tasks. In conclusion, while all three designs show potential in detecting schizophrenia, the 3D-CNN model is the most accurate. Its capacity to handle 3D sMRI data improves feature representation, resulting in improved diagnostic performance.

**Fig. 18:**
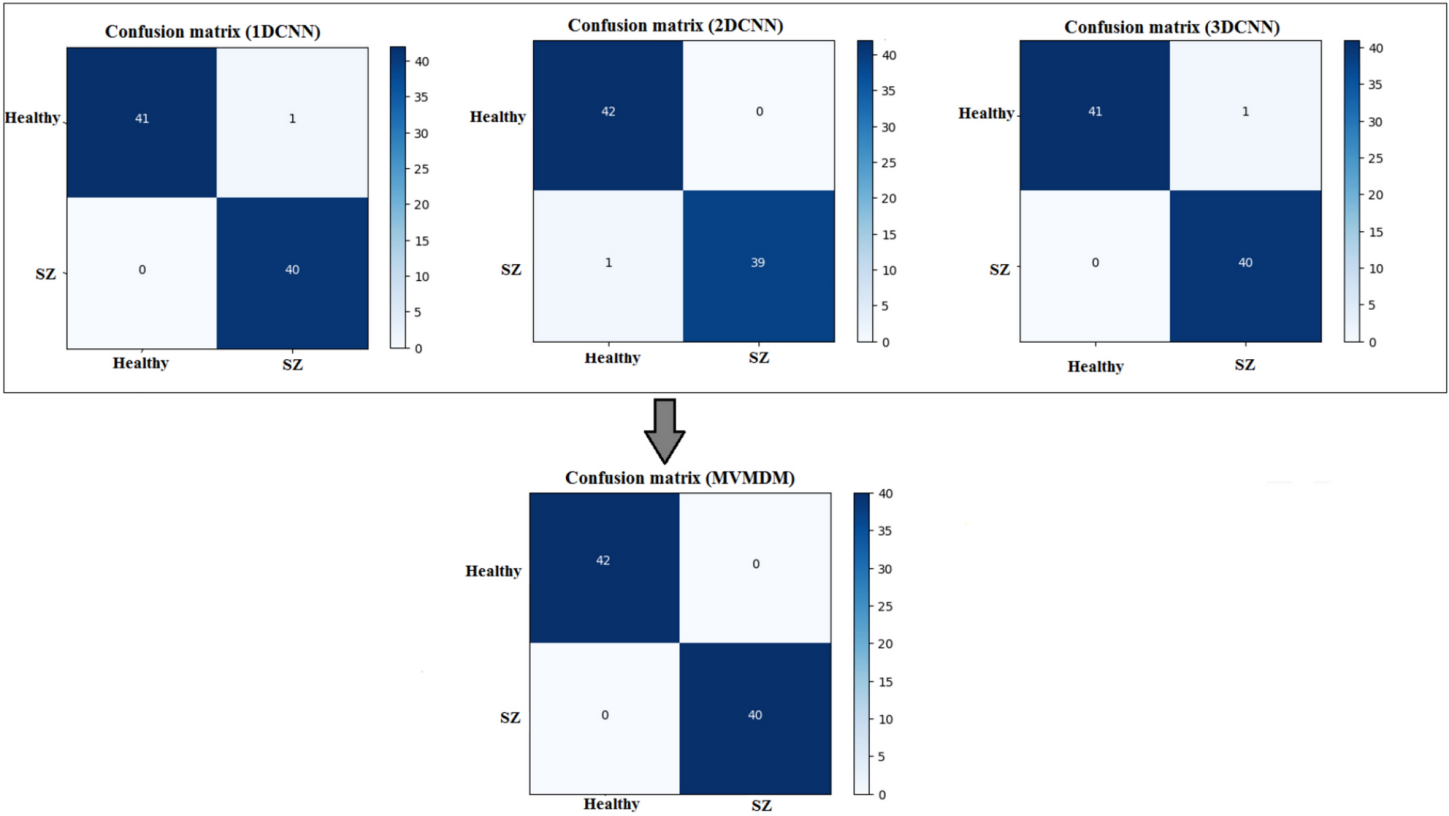
Confusion matrix analysis of MVMDM for the test data.

**Fig. 19:**
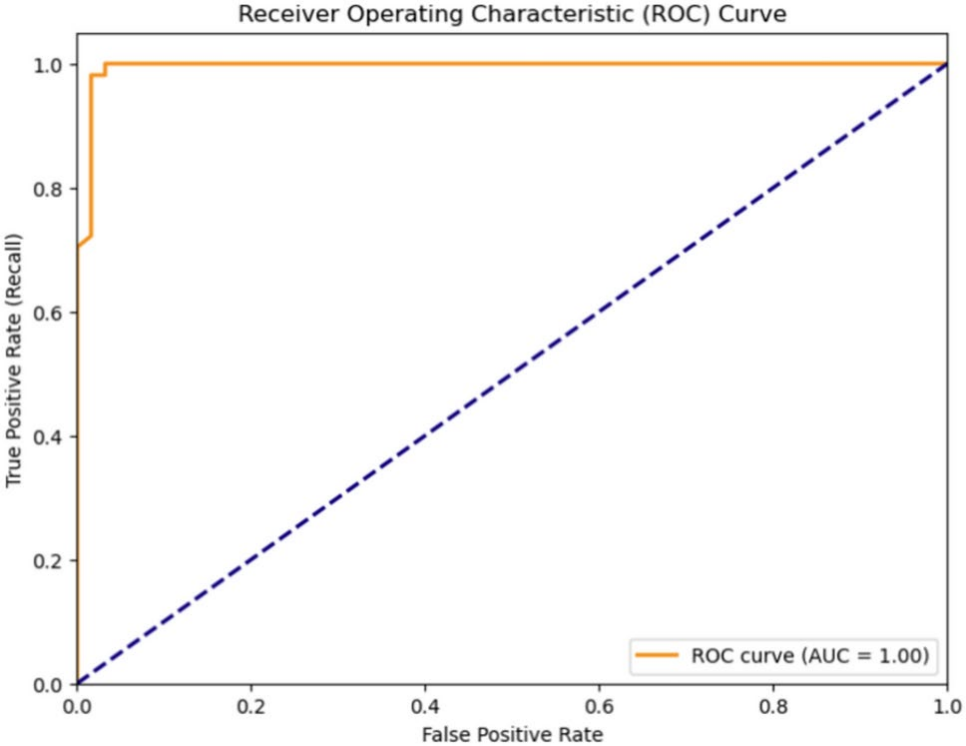
ROC curve of MVMDM model.

**Table 13:** Performance analysis of MDCNNs and MVMDM in prediction. Where $\times$ represents a healthy subject, whereas $\checkmark$ implies schizophrenia.

Test data	1DCNN	2DCNN	3DCNN	MVMDM
1	$\times$	$\times$	$\times$	$\times$
2	$\times$	$\checkmark$	$\checkmark$	$\checkmark$
3	$\checkmark$	$\checkmark$	$\checkmark$	$\checkmark$
4	$\times$	$\checkmark$	$\times$	$\times$
Testing accuracy	99.75%	99.51%	99.69%	100%

The confusion matrices, presented in Fig. 18, for the MDCNNs models, and MVMDM technique, show clearly how they perform in identifying schizophrenia versus healthy controls. The 1D-CNN and 3D-CNN models had one false positive and no false negatives, meaning the accuracy was high. The 2D-CNN model resulted in one false negative and no false positives. The approach MVMDM, combining the predictions of the three models, achieved perfect accuracy with no misclassifications In a scenario where each model receives a single sMRI input, the 1D-CNN may misidentify a healthy individual as having schizophrenia, whereas the 2D-CNN and 3D-CNN models correctly identify the individual as healthy. The MVMDM approach would correctly classify this subject as healthy by aggregating the predictions. Table 13 compares the performance of MDCNN and MVMDM on four test data. The results are displayed with ticks ($\checkmark$) and crosses ($\times$). A tick represents a healthy person, whereas a cross implies schizophrenia. In Test Data 2, the 1DCNN shows schizophrenia, whereas the 2DCNN and 3DCNN show healthy, and the MVMDM model delivers healthy as prediction. In the scenario of Test Data 4, 1DCNN and 3DCNN predict schizophrenia, but 2DCNN predicts healthy, whereas MVMDM predicts schizophrenia. The performance of the proposed model, MVMDM is also evaluated using receiver operating characteristics (ROC) curve and is presented in Fig. 19. In this paper, the ensemble strategy utilizes the strengths of MDCNNs to improve generalization performance in SZ classification.

**Fig. 20:**
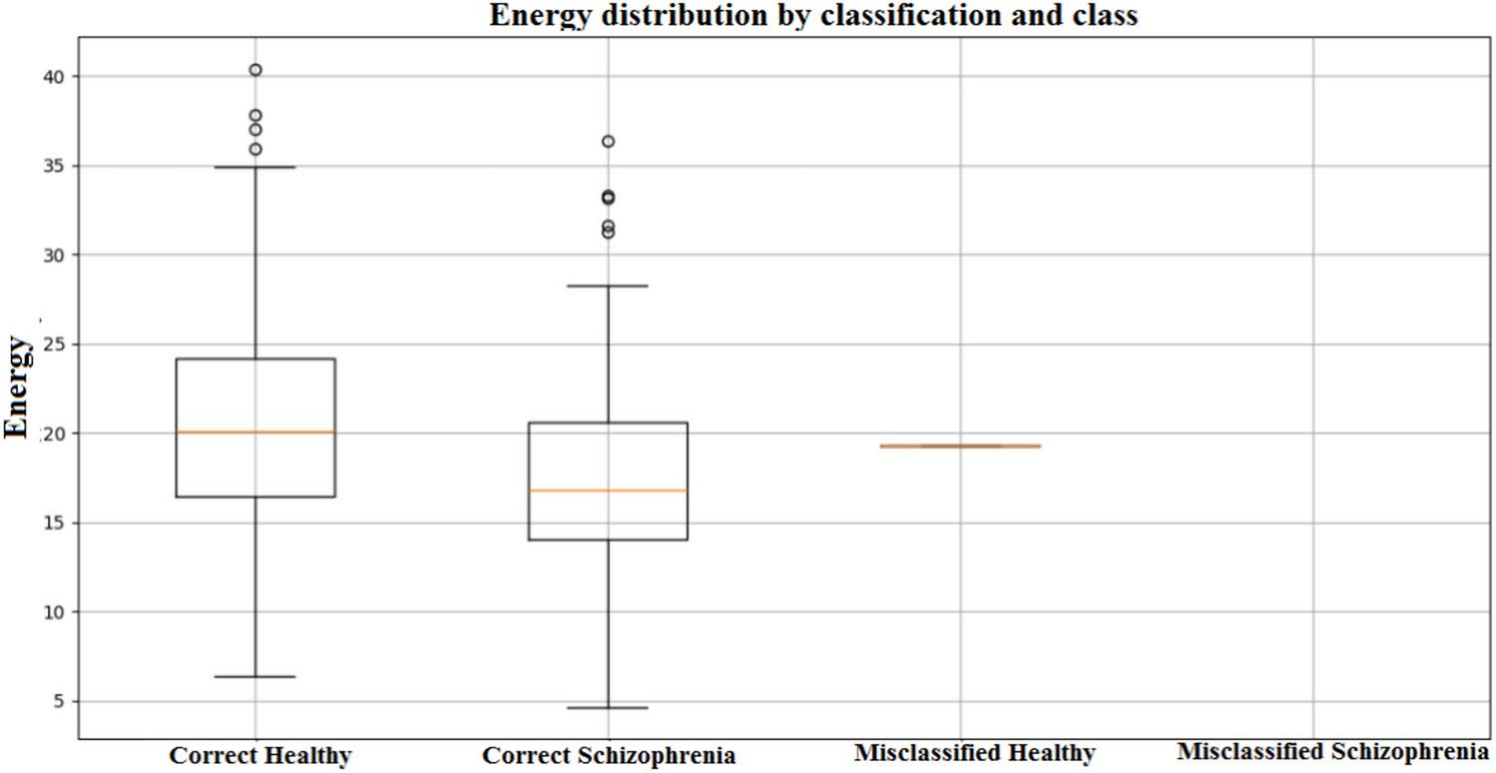
Energy distribution of correctly classified and misclassified labels for 1DCNN.

Figure 20 displays the energy distribution of sMRI samples that are classified into four categories using 1D CNN: correct schizophrenia, correct healthy, misclassified schizophrenia, and misclassified healthy. Whereas samples with schizophrenia exhibit a narrow range with lower medians, healthy individuals exhibit a wider range with higher median values for correctly categorized samples. The model finds it easier to classify healthy samples since their energy properties are more diverse, while those of schizophrenia samples are more consistent. A healthy person who is mistakenly diagnosed with schizophrenia, the misclassified sample, has an energy value that is more in line with the spectrum of schizophrenia. This misconception may be caused by elements like noise in the sMRI data or minute anatomical differences in the incorrectly categorized sample. The confusion matrix and related metrics are important for determining the effectiveness and constraints of the 2D CNN model when applied to classify SZ from sMRI images. Even though the classification performance is good in general, the misclassified sample has unique features that call for more research. In particular, the average sharpness of the correctly classified samples is 0.0093, which is much higher than the misclassified sample sharpness equal to 0.0082. This means the performance of the model in correctly classifying specific samples may depend on the quality of the images, particularly sharpness. Besides, the model is not too confident about its prediction because the confidence score of the misclassified sample is 0.5149, close to the 0.5 decision threshold. The above uncertainty is also brought out by the -0.0871 confidence gap between correctly classified samples and the wrongly classified sample. The outcome shows that factors such as low-quality images or ambiguous anatomical features might affect misclassification. The misclassified sample of 3DCNN model analysis is meant to extract some very important conclusions regarding effectiveness as well as limitations of the model. Though the confidence score of the misclassified sample at 0.6207 is above the decision threshold at 0.5, it is still far below the average confidence score obtained by successful classification samples. The gap between confidence of -0.1841 made the model more uncertain about the wrong predictions rather than correct ones. Also, while the average sharpness of the correctly classified samples is 0.0098, the misclassified sample’s sharpness is 0.0080. This means that the image quality of the misclassified sample may be poorer, which might make it harder for the model to find characteristic features.

## Comparative analysis

### Performance comparison of MVMDM with existing models across multiple metrics

We compared the proposed method with other methods that have used the same datasets in this experiment. The customized 1D-CNN classifier, using the energy features extracted, showed outstanding results of 98.1% accuracy. The 2D-CNN classifier achieved 99.5% accuracy. The 3D-CNN attained an accuracy of 99.6%. It is thus a very all-embracing comparison of many methods in use within the domain of sMRI research. It was thus established that the proposed MVMDM method would outperform the existing ones in classification performance.

**Table 14:** Performance analysis of MVMDM with other models.

Models	Accuracy (%)	Precision (%)	Sensitivity (%)	Specificity (%)	F1 score (%)
3D-CNN^18^	88.5	86.0	92.0	85.1	89.3
DNN^60^	85.7	81.2	89.2	82.0	80.0
SE-VGG-11BN^61^	93.3	94.4	94.9	94.4	94.5
DBN^62^	90.0	93.3	87.5	92.8	90.3
LSTM	93.7	90.8	94.9	92.7	92.8
ImageNet	96.9	96.6	98.2	95.1	97.4
1D-CNN	99.7	100	99.4	100	99.7
2D-CNN	99.5	98.8	100	99.1	99.4
3D-CNN	99.6	99.2	100	99.4	99.6
MVMDM	100	100	100	100	100

Table 14 provides a comparison of performance between various models including 3D-CNN^18^,DNN^60^, SE-VGG-11BN^61^, DBN^62^, LSTM, ImageNet, MDCNN models and proposed MVMDM model. Accuracy, precision, sensitivity, specificity, and F1 Score have been used to carry out the performance comparison. From established models, DNN was with an accuracy of 85.7%, then SE-VGG-11BN attained an accuracy of 93.3%, while DBN has an accuracy of 90.0%. These models have competitive performance in all criteria. The 3D-CNN model has achieved an accuracy of 88.5%, which shows its usefulness in classification tasks. LSTM has also performed well with an accuracy score of 93.7%. Table 13 hereby explains three architectures of the MDCNNs. The models have excellent performances because the accuracy scores of those models range from 99.5% to 99.7%. The accuracy of the 1D-CNN is 99.7% that shows excellent performance for a classification task. At the last, the MVMDM model outperformed other models because it achieves excellent perfect scores of 100% for accuracy, precision, sensitivity, specificity, and F1 Score. This shows that the MVMDM model outperforms all other models tested in this study. The model’s performance is tested with different datasets to look at the issue of overfitting.

### Comparative performance analysis of transforms (DWT, FT, HT, and HHT) on neuroimaging datasets

**Table 15:** Performance analysis on DWT, FT, HT, and HHT.

Transform	COBRE (74, 71)	Open fMRI (42, 58)	UCLA (119, 48)
**Accuracy (%)**			
Wavelet transform	CA=94.50 CV=98.52 CH=95.08 CD=97.00	CA=90.59 CV=96.45 CH=98.58 CD=97.14	CA=95.29 CV=98.23 CH=97.08 CD=96.82
Fourier transform	Magnitude=80.11 Phase=75.74	Magnitude=78.9 Phase=80.5	Magnitude=71.24 Phase=82.35
Hilbert transform	Magnitude=78.2 Phase=61.9	Magnitude=73.64 Phase=60.07	Magnitude=72.36 Phase=65.29
Hilbert-Huang transform	IMFs=69.4	IMFs=71.8	IMFs=70.4

For the classification of structural MRI images into schizophrenia and healthy control, the DWT consistently outperforms other approaches, achieving approximately 98% accuracy across multiple datasets (COBRE, Open fMRI, and UCLA) as presented in Table 15. Its ability to detect both high and low-frequency spatial features makes it suitable for detecting small structural alterations which are indicative of schizophrenia. In contrast, the Fourier Transform excels at assessing frequency elements but struggles to capture delicate spatial information, which is critical for differentiating schizophrenia. The Hilbert Transform provides insights into the image phase and magnitude, excelling in magnitude analysis across multiple datasets, but its phase analysis is variable, particularly. The Hilbert-Huang Transform divides images into intrinsic mode functions and has certain advantages when processing non-linear brain signals, but it is not as effective as other transforms. In general, the DWT is the most suitable for distinguishing schizophrenia from healthy subjects.

**Fig. 21:**
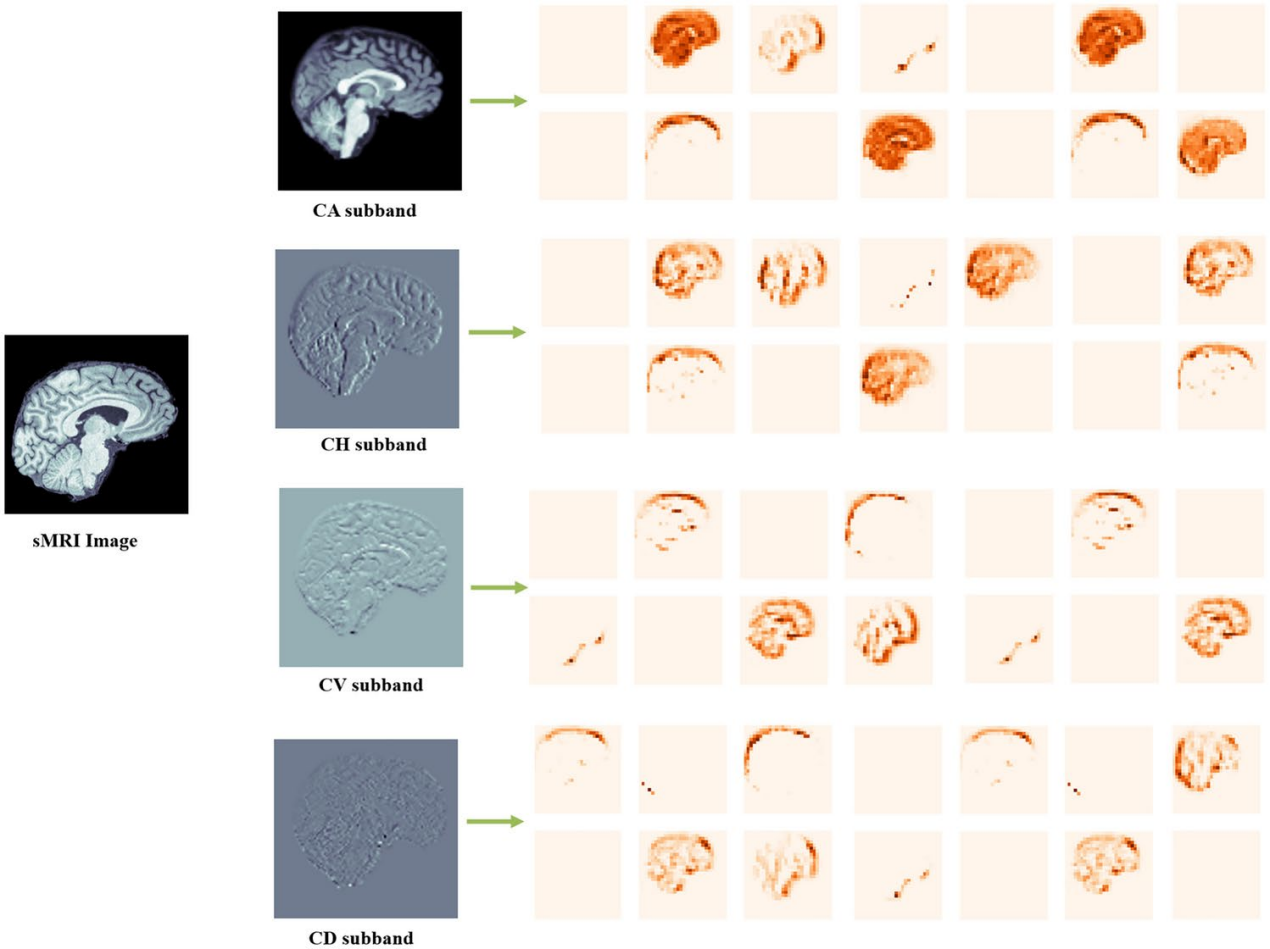
Visualization of wavelet transformed images and feature maps.

The Fourier Transform, Hilbert Transform, and Hilbert-Huang Transform are suboptimal for representing localized features in sMRI images than DWT. FT breaks down signals into frequency elements but overlooks spatial details. HT offers phase data while not preserving spatial configuration. HHT, a time-frequency approach, provides detailed frequency information but does not support spatial awareness. In contrast, the wavelet technique preserves both frequency and spatial details, allowing for more targeted investigation of brain regions. The feature maps, along with visualization methods like Grad-CAM, shows discrete wavelet transform’s ability to classify schizophrenia using sMRI images. The feature maps are presented in Fig. 21.

In conclusion, the importance of frequency components for SZ classification using sMRI analysis is substantial, as both low and high frequencies provide crucial structural data. The DWT is particularly effective for schizophrenia classification because it can identify both large and detailed features via multi-resolution analysis, whereas alternatives such as the Fourier Transform and Hilbert Transform concentrate too exclusively on frequency or phase data, or do not adequately capture the multi-scale characteristics of brain structures. The DWT’s efficiency and adaptability in examining both frequency and spatial elements render it the most suitable decomposition method for the classification of schizophrenia from healthy subjects in sMRI image evaluation.

## Generalization of the MVMDM

**Fig. 22:**
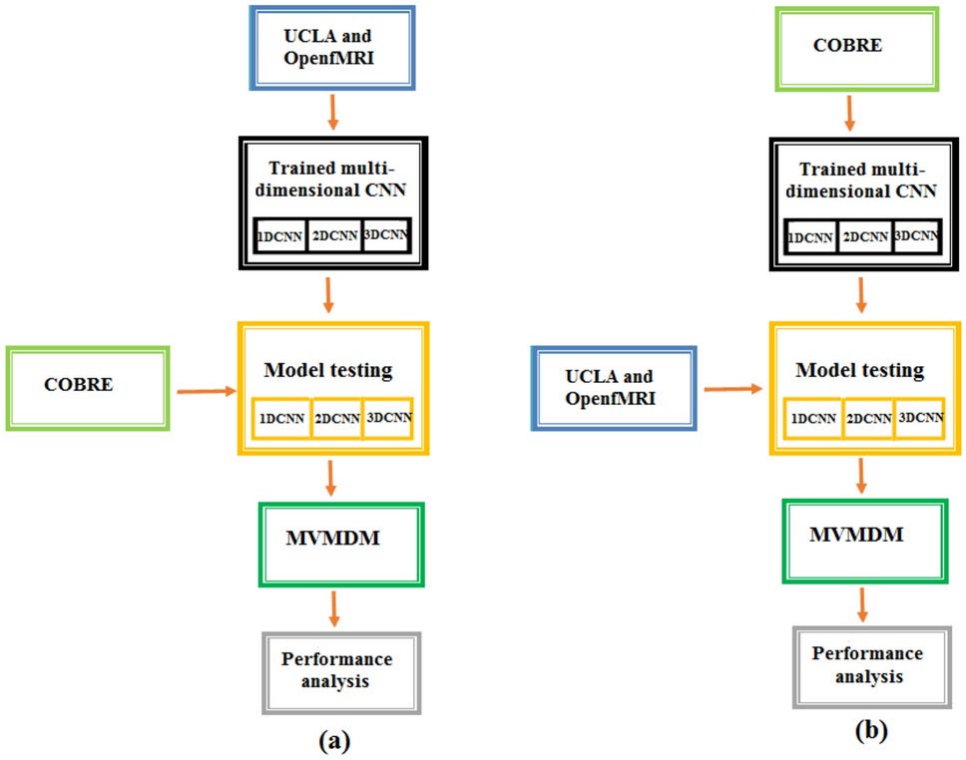
Generalization of MVMDM Model (**a**) UCLA and OpenfMRI for training and COBRE for testing (**b**) COBRE for training and UCLA and OpenfMRI for testing.

The performance of deep learning models can be assessed by generalization which can critically evaluate the model using different datasets for training and testing. In this study, we consider two scenarios: COBRE dataset (74 healthy and 71 schizophrenia) for training UCLA and OpenfMRI dataset (160 healthy and 106 schizophrenia) for testing and vice versa is illustrated in Fig. 22. The MDCNNS architectures achieve average generalization accuracies of 93.2%, 95.8%, and 98.0%, respectively, while the proposed model achieves an accuracy of 98.9%.

**Table 16:** Generalization analysis using COBRE dataset for training and UCLA and OpenfMRI dataset for testing.

Model	Accuracy (%)	Precision (%)	Sensitivity (%)	Specificity (%)	F1-score (%)
**CA coefficient**					
1D-CNN	85.33	88.50	72.84	93.75	79.79
2D-CNN	89.01	66.66	81.81	98.75	85.71
3D-CNN	84.93	77.50	93.93	77.50	84.93
**CH coefficient**					
1D-CNN	84.96	80.55	82.07	86.87	81.30
2D-CNN	**94.36**	**98.92**	**86.79**	**99.37**	**92.46**
3D-CNN	74.17	67.44	68.23	78.12	67.83
**CV coefficient**					
1D-CNN	80.07	76.23	72.64	85.00	74.39
2D-CNN	88.72	95.23	75.47	97.50	84.21
3D-CNN	**98.13**	**98.11**	**97.22**	**98.81**	**97.74**
**CD coefficient**					
1D-CNN	**94.73**	**96.93**	**89.62**	**98.12**	**93.13**
2D-CNN	90.22	100	75.47	100	86.02
3D-CNN	80.55	83.33	78.94	82.35	81.08
**Proposed model**					
MVMDM	98.49	98.11	98.11	88.75	98.11

Table 16 Compares the performance of MDCNNs models and the proposed MVMDM model. The evaluation employed the COBRE dataset for training and the UCLA and OpenfMRI dataset for testing, on four DWT subbands. The 1D-CNN excels using CD subband, correctly detecting true positive cases while minimizing false positives, false negatives, and true negatives. The 2D-CNN excels well with the CH subband, effectively identifying TP cases while keeping a balance with FP, FN, and true negative (TN) examples. The 3D-CNN utilizes the CV subband, which provides good precision in the detection of positive true cases and minimizes FP and FN cases; it also classifies the TN cases appropriately. Hence, the findings led to the development of the MVMDM model, that uses a max-voting ensemble method to combine the powers of MDCNNs The MVMDM model performed well in recognizing TP cases, minimizing FP and FN instances, and correctly classifying TN cases. This combination produced a strong and successful model for schizophrenia classification, demonstrating the advantages of combining the characteristics of MDCNN architectures.

**Table 17:** Performance metrics: UCLA and OpenfMRI dataset for training and COBRE dataset for testing.

Model	Accuracy (%)	Precision (%)	Sensitivity (%)	Specificity (%)	F1-score (%)
**CA coefficient**					
1D-CNN	84.90	86.36	79.16	89.65	82.60
2D-CNN	89.84	96.47	77.35	98.12	85.86
3D-CNN	96.74	94.11	98.77	96.47	96.96
**CH coefficient**					
1D-CNN	86.38	82.71	81.70	89.31	82.20
2D-CNN	**97.24**	**98.55**	**95.77**	**98.64**	**97.14**
3D-CNN	74.17	67.44	68.23	78.17	67.83
**CV coefficient**					
1D-CNN	69.82	66.10	72.22	67.74	69.02
2D-CNN	88.96	100	77.46	100	87.30
3D-CNN	**97.98**	**95.81**	**100**	** 96.11**	** 97.99**
**CD coefficient**					
1D-CNN	**91.72**	**95.38**	** 87.32**	** 95.94**	**91.17**
2D-CNN	82.06	73.68	98.59	66.21	84.33
3D-CNN	90.48	92.00	82.83	83.29	90.87
**Proposed model**					
MVMDM	99.31	98.61	100	98.64	99.30

Table 17 displays performance values for MDCNNs, including MVMDM, trained on the UCLA and OpenfMRI datasets and tested on the COBRE dataset. The evaluation concentrated on four DWT subbands. Building on the performance of MDCNNs using different DWT subbands, the proposed MVMDM model uses a max-voting technique. This ensemble strategy improves overall performance, resulting in a model that minimizes FP and FN cases, and correctly classifying TN cases. The MVMDM model’s ability to capitalize on the unique capabilities of each CNN architecture results in a commendable performance in classification tasks.

**Table 18:** Confusion matrix analysis for healthy and schizophrenia using COBRE dataset for training and UCLA and OpenfMRI dataset for testing.

	1D-CNN		2D-CNN		3D-CNN		MVMDM	
Classes	Healthy	SZ	Healthy	SZ	Healthy	SZ	Healthy	SZ
Healthy	157	3	159	1	157	3	158	2
SZ	11	95	14	92	2	104	2	104

Table 18 shows confusion matrices for MDCNNs and the proposed MVMDM model for distinguishing healthy persons and those with schizophrenia, trained on the COBRE dataset and tested on the UCLA and OpenfMRI datasets. The 1D-CNN model properly classified 157 healthy persons and 95 people with schizophrenia, with three and eleven misclassifications, respectively. The 2D-CNN model accurately recognized 159 healthy persons and 92 people with schizophrenia, with 1 and 14 misclassifications, respectively. The 3D-CNN model accurately classified 157 healthy people and 104 schizophrenia patients, with three and two misclassifications, respectively. The MVMDM model outperformed the others, properly categorizing 158 healthy persons with only two misclassifications while reaching perfect accuracy for schizophrenia. These findings demonstrate the MVMDM’s better accuracy and efficiency in discriminating between healthy and schizophrenic patients.

**Table 19:** Confusion matrix analysis for healthy and schizophrenia using UCLA and OpenfMRI dataset for training and COBRE dataset for testing.

	1D-CNN		2D-CNN		3D-CNN		MVMDM	
Classes	Healthy	SZ	Healthy	SZ	Healthy	SZ	Healthy	SZ
Healthy	71	3	73	1	74	0	73	1
SZ	9	62	3	68	3	68	0	71

Table 19 presents the confusion matrix for distinguishing between healthy and schizophrenia participants, using MDCNNs and the proposed MVMDM Using the UCLA and OpenfMRI datasets for training and the COBRE datasets for testing. The 1D-CNN model accurately categorized 71 healthy individuals and 62 schizophrenia patients, with 3 and 9 misclassifications, respectively. The 2D-CNN improved this by properly recognizing 73 healthy persons and 68 people with schizophrenia, with only one and three misclassifications, respectively. The 3D-CNN had 100% accuracy for 74 healthy individuals and 68 correct classifications for schizophrenia, with three misclassifications. The MVMDM model surpassed the others by properly classifying 73 healthy persons with only one misclassification and obtaining perfect accuracy for schizophrenia. These findings highlight the MVMDM’s capability to distinguish between healthy and schizophrenic patients.

**Table 20:** Mean accuracy and Mean SD.

Model	Mean accuracy	Mean standard deviation (SD)
1DCNN	0.975	0.010
2DCNN	0.978	0.012
3DCNN	0.992	0.005

**Fig. 23:**
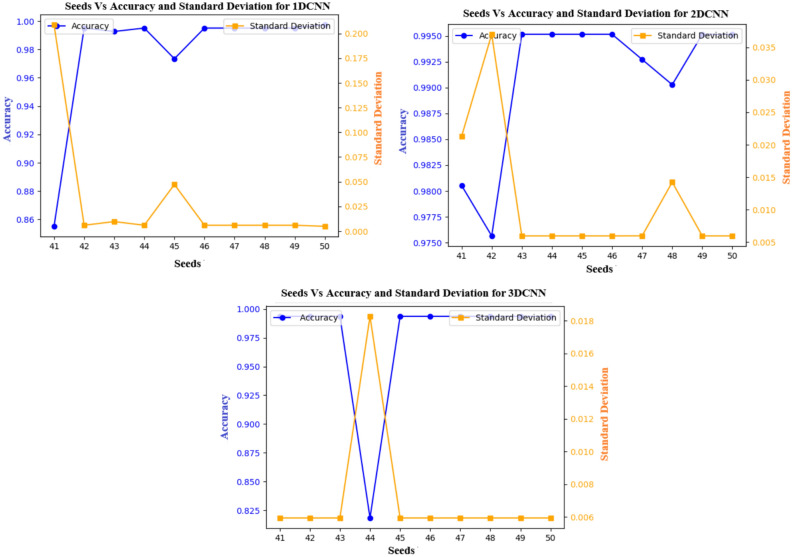
Different seeds Vs accuracy and standard deviation.

**Fig. 24:**
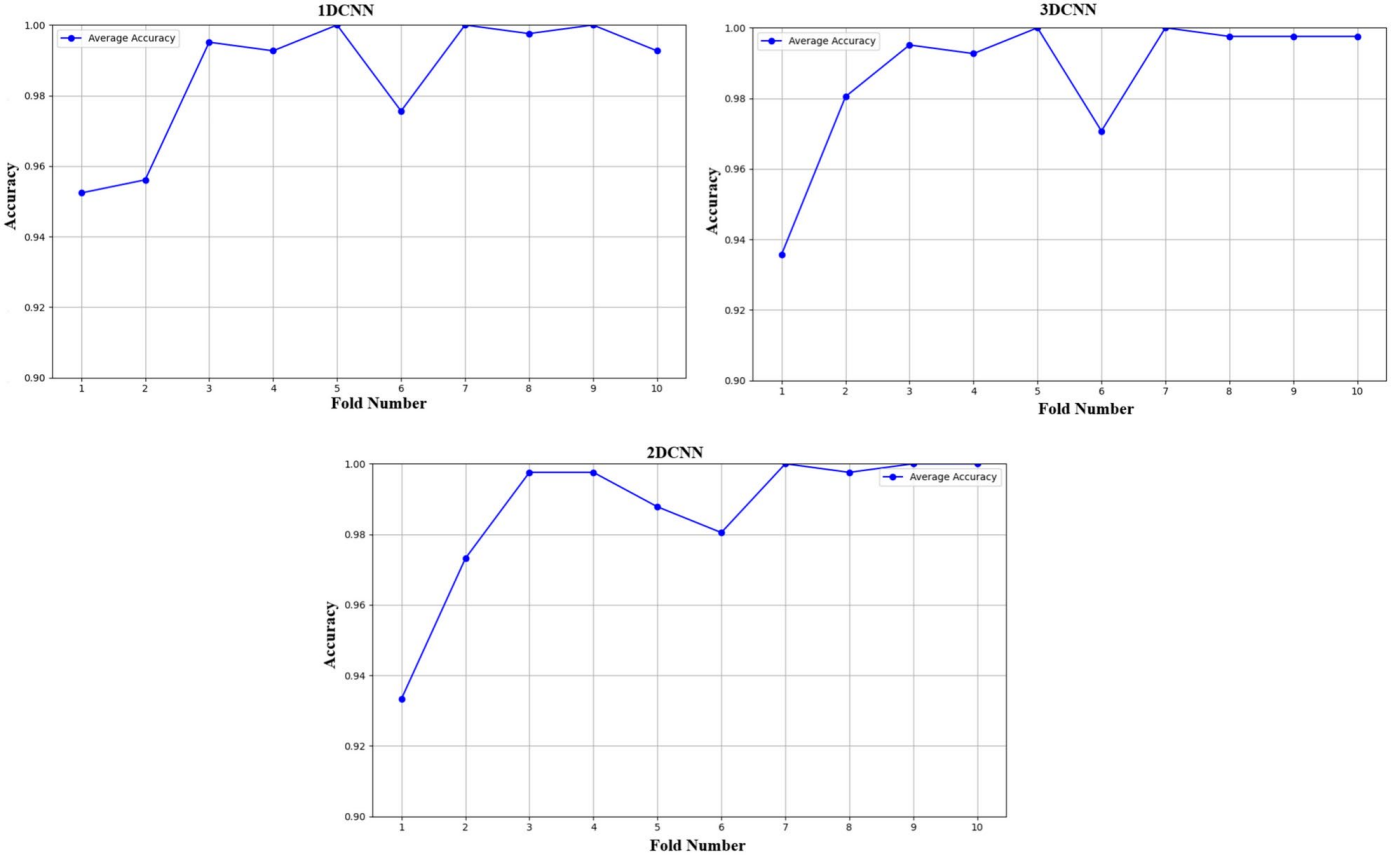
Different fold Vs accuracy.

To assure the stability of the proposed model, the effects of various random seeds, and k-fold cross-validations are investigated. The models (1D-CNN, 2D-CNN, and 3D-CNN) were tested under 10 different random seed settings from 41 to 50 illustrated in the Fig. 23 using 10-fold cross-validation illustrated in the Fig. 24, with mean accuracy and standard deviation (SD). Despite changes in random seeds, the 1D-CNN’s mean accuracy remains steady, showing only slight deviations and a low standard deviation. The 2D-CNN shows steady improvement throughout folds, with minor variations, leading to a marginally higher SD than the 1D-CNN. With nearly constant accuracy across all folds and the lowest SD, the 3D-CNN yields the most reliable results, proving its resilience to cross-validation and random seed changes. The conclusion that the models generalize well and produce reliable prediction on previously unseen data. Stability and reproducibility of the models are statistically confirmed by the mean accuracy and standard deviation presented in the table 20. The low SD for 3D-CNN demonstrates its improved capacity to grasp spatial dependencies in 3D volumetric data, which increases its resistance to changes in initialization and random seeds. In summary, the statistical reliability, repeatability, and durability of the experimental results are confirmed by the combination of k-fold cross-validation and random seed evaluations.

## Limitations and future work

The proposed method ensembles the classification outputs from multidimensional models. The features are extracted from DWT coefficients that give emphasis to multiscale high frequency details. However, the proposed model has a few limitations in the form of computational complexity. The multidimensional CNNs have different number of tunable parameters which will increase the duration of model training. Feature extraction in DWT domain also increases computational complexity due to wavelet decomposition. However, the first level decomposition reduces the amount of data used for model training in all the three CNN models. The ensemble strategy leads to overfitting that decreases the accuracy in a real-time prediction due to less generalizability of the model. The prediction accuracy decreases when the model is generalized using different datasets for training and testing. We acknowledge that there is currently limited literature directly linking DWT sub-bands to schizophrenia, and we appreciate your suggestion to strengthen the biological interpretability. In the revised paper, we highlight that the selection of DWT sub-bands was guided by theoretical considerations and prior research in image processing and neuroimaging, which suggest that wavelet transform-based features can capture meaningful patterns in brain activity and structural changes. The selection of subbands and relevant feature extraction in wavelet domain is decided based on the experimental and ablation study using supervised learning. sMRI would not be able to capture several key aspects of the illness, such as altered neural network interactions or transient dysfunctions^63^. Structural changes such as ventricular enlargement or reductions in gray matter may be observed on sMRI but fails to do so for a few cases. Additional biomarkers, such as fMRI^63^ or clinical data, can enhance the diagnostic accuracy of the suggested model. The functional activity of the brain acquired by fMRI can supplement sMRI by helping to comprehend brain connections and neural network abnormalities that structural imaging alone may not detect. The dataset availability of sMRI and fMRI brain scans for the same subjects is a major factor to rely on one modality for SZ classification. A common trade-off between number of features for training/testing and performance is another influencing factor for one modality. The future scope of this work can deal with SZ classification using both functional and structural MRI data. The complexity due to multidimensional model training could also be replaced by other ensemble strategies. The overfitting problem due to simple max-voting strategy can be investigated further using multidimensional feature concatenation. The proposed model is generalized using three datasets in which the overfitting problem is avoided. When the model is generalized using multiple datasets, the model performance could be optimized using Bayesian optimization.

## Conclusion

Schizophrenia is a complicated brain condition marked by symptoms such as hallucinations, delusions, disordered thinking, and a flat affect, which indicate substantial structural and functional brain abnormalities. sMRI is important in the detection of schizophrenia because it provides specific brain images that allow physicians to find structural anomalies. In this paper, we have proposed a max-voting ensemble method using MDCNNs. The MDCNNs were trained using selected DWT subbands. Energy feature-based 1DCNN was experimented on the CD subband, fusion-based 2DCNN was experimented on the CH subband, and volumetric-based 3DCNN was experimented on the CV subband. The proposed method, MVMDM, was tested on COBRE, UCLA and OpenfMRI datasets and the performance was analyzed by accuracy, precision, sensitivity, specificity, and F1-score. The observed MVMDM results prove the role of the ensemble approach on the MDCNNs. It is observed that the subjects with schizophrenia have lower gradient magnitudes of pixel values across brain regions in sMRI brain scan. These changes are observed in prefrontal cortex, temporal cortex, insula, cingulate cortex, basal ganglia, hippocampus, amygdala, and ventricular structures when compared. As a result, this paper employs multidimensional CNNs tailored to distinct subbands of wavelets, accurately captures the multidimensional anomalies that present in these locations. The use of 1D, 2D and 3D CNNs will allow for a more in-depth investigation of these abnormalities, resulting in a better understanding of the pathophysiology of schizophrenia and supporting future diagnostic and therapeutic advances. The multidimensional CNN models also work on wavelet coefficients that highlight textural changes, fine, coarse details and edges along four directions. The method was generalized using two datasets and the performance was analyzed. Specifically, the MDCNNS achieves an average accuracy of 93.2%, 95.8%, and 98.0%. The proposed MVMDM model achieved an overall accuracy of 98.9%, underscoring the enhanced predictive abilities of our approach for schizophrenia classification. The MVMDM method also reduces FP and TN cases from the MDCNN outputs, making it more prominent. Maximum voting on the outputs of the best-performing MDCNNs is anticipated to result in very accurate results for Schizophrenia classification with new sMRI data, enhancing trust in our approach’s predicting abilities.

## Data Availability

The datasets generated and/or analyzed during the current study are available in the following repositories: (i) UCLA Consortium for Neuropsychiatric Phenomics LA5c Study database - https://openfmri.org/dataset/ds000030/ (ii) OpenfMRI dataset - https://openfmri.org/dataset/ds000115/ (iii) COBRE dataset - https://fcon_1000.projects.nitrc.org/indi/retro/cobre.html.
